# The association of screen time and the risk of sleep outcomes: a systematic review and meta-analysis

**DOI:** 10.3389/fpsyt.2025.1640263

**Published:** 2025-12-17

**Authors:** Xin He, Bei Pan, Ning Ma, Dan Li, Weize Kong, Qian Liu, Xiaowei Liu, Xiaoman Wang, Xiyuan Deng, Kehu Yang

**Affiliations:** 1 Centre for Evidence-Based Social Science/Center for Health Technology Assessment, School of Public Health, Lanzhou University, Lanzhou, China; 2 Evidence-Based Medicine Center, School of Basic Medical Sciences, Lanzhou University, Lanzhou, China, Lanzhou University, Lanzhou, China; 3 Gansu Key Laboratory of Evidence-Based Medicine, Lanzhou University, Lanzhou, China; 4 The First Medical College of Lanzhou University, Lanzhou, China; 5 Department of Prenatal Diagnosis Center, Gansu Provincial Maternity and Child-care Hospital, Lanzhou, China

**Keywords:** screen time, sleep duration, insomnia, systematic review, meta-analysis

## Abstract

**Introduction:**

Screen time has become increasingly prevalent in modern life and may influence various health outcomes, including sleep patterns. Previous meta-analyses examining the relationship between screen time and sleep have been limited by incomplete population coverage and insufficient consideration of potential effect modifiers. To address these gaps, we conducted a comprehensive meta-analysis to investigate the association between screen time and sleep outcomes across diverse populations.

**Methods:**

Two independent reviewers screened studies and extracted data following a pre-registered protocol. Standardized coefficients (β) and odds ratios (OR) were used to quantify effect sizes. Random-effects meta-analyses were conducted using STATA 17.0, with subgroup analyses performed to explore effect modifiers.

**Results:**

We included 21 cohort studies with 548,338 participants. Each additional hour of daily screen time was associated with approximately 3 to 5 minutes shorter total sleep duration (β = −0.05, 95% CI: −0.08 to −0.03) in 11 studies reporting continuous outcomes, and with a higher risk of short sleep in nine studies reporting binary outcomes (OR = 1.25, 95% CI, 1.08 to 1.40). Subgroup analyses found no significant effect modification by age, region, short sleep definition or follow-up duration (all P _interaction_ > 0.05). However, for binary outcomes, the association between screen time and short sleep differed significantly across countries (P _interaction_ = 0.004). For other sleep outcomes, longer screen time was associated with increased risk of insomnia symptoms (β = 0.41, 95% CI, 0.18 to 0.63), delayed bedtime (13.2 minutes delay per hour of screen time), and difficulty initiating sleep (OR = 3.05; 95%CI: 1.51 to 6.24).

**Conclusion:**

This systematic review demonstrates a robust association between increased screen time and adverse sleep outcomes, with adolescents showing particular vulnerability. These findings underscore the importance of screen time management in sleep health promotion and suggest the need for age-specific interventions. Future research should focus on establishing causal relationships and developing evidence-based guidelines for optimal screen use across different age groups.

**Systematic review registration:**

https://www.crd.york.ac.uk/prospero/, identifier CRD42023476130.

## 1 Introduction

Adequate sleep duration plays a vital role throughout the human lifespan. Short sleep duration has been associated with increased risk of obesity, while both insufficient and excessive sleep have been linked to adverse health outcomes including increased mortality, type 2 diabetes (1), and hypertension (2). International guidelines (3) recommend age-specific sleep durations: 10–14 hours for preschoolers (3-5y), 7–9 hours for young adults (18-25y) and adults (26-64y), and 7–8 hours for older adults (>65y) (3). However, insufficient sleep and sleep disturbance have become increasingly common among youth and adolescents worldwide (4). Poor sleep can lead to multiple adverse effects including depression, excessive daytime sleepiness, and metabolic dysfunctions (5–8).

The digital revolution has fundamentally transformed daily life (9). Today’s children and adolescents spend increasing amounts of time on electronic devices such as phones, tablets, and computers (10). This trend is concerning as behavioral patterns established during childhood often persist into adulthood (11). The 2016 Canadian 24-hour Movement Guidelines recommend limiting screen time to less than 2 hours per day for children and adolescents (12). However, current research indicates that only 16.8-41.6% of young people meet these guidelines (13). Moreover, emerging evidence indicates that higher engagement in sedentary screen activities, such as television viewing, computer work, and internet use, is associated with poorer sleep outcomes in adults (14).Unfortunately, formal recommendations for screen time use of adults are scarce and vary widely across organizations.

Several systematic reviews and meta-analyses have explored the relationship between screen time and sleep outcomes. Janssen study (15)reported that screen time is associated with poor sleep outcomes in infants and schoolers. Carter (16) showed that strong and consistent evidence of an association between access to or the use of devices and reduced sleep quantity for children and adolescents of school age between 6 and 19 years. Li (17) suggested that excessive screen time was associated with overweight/obesity and shorter sleep duration among toddlers and preschoolers. Poitras (18) found that these findings continue to support the importance of minimizing screen time for disease prevention and health promotion in the early years, but also highlight the potential cognitive benefits of interactive non-screen-based sedentary behaviors such as reading and storytelling. Vallance (19) thought that with respect to sleep outcomes, context-specific sedentary behaviors may be more important than overall sedentary time. Hale (20) conducted a systematic literature review study for children and adolescents. The study conducted that screen time is adversely associated with sleep outcomes in most of the studies reviewed.

However, key limitations in the existing reviews necessitate the present study. (1) Most analyses focus on children and adolescents, providing limited coverage of adults and older adults.(2) Existing reviews have not comprehensively examined potential effect modifiers such as socioeconomic status and geographic regions. (3) Many reviews combine cross-sectional and longitudinal designs, impeding temporal precedence and increasing vulnerability to reverse causation. These gaps motivate a design-restricted synthesis that harmonizes effect metrics and systematically explores modifiers.

To address these limitations, we conducted a PRISMA-guided systematic review and meta-analysis that exclusively includes cohort studies, reflecting the stronger capacity of longitudinal designs to establish exposure–outcome ordering and reduce selection bias (21). We prespecified harmonized effect measures for binary and continuous outcomes, prioritized clinically interpretable units where feasible, and planned subgroup and sensitivity analyses to explore heterogeneity by age, region, and socioeconomic status.

Therefore, our objectives were to: (1) quantitatively synthesize the association between screen time and total sleep duration (primary outcome) across different populations; (2) investigate potential effect modifiers, including age, region, and socioeconomic status; and (3) evaluate associations with secondary sleep outcomes, such as insomnia symptoms and sleep timing (bedtime and wake time).

## 2 Methods

### 2.1 Protocol and registration

The protocol of this study was registered in the International Prospective Register of Systematic Reviews (PROSPERO; Registration: No. CRD42023476130). This systematic review and meta-analysis were performed according to the Preferred Reporting Items for Systematic Reviews and Meta-Analysis (PRISMA) Statements (22). All inclusion criteria, pre-specified primary outcomes, planned subgroups, and overall analysis strategy remain consistent with the registered protocol. The only modification pertains to the risk of bias assessment, which was updated from a modified NOS approach to the ROBINS-E tool to provide a more detailed, domain-specific evaluation of bias in exposure measurement, outcome assessment, and participant selection. This change does not affect outcomes or planned analyses but ensures that GRADE assessments accurately reflect the methodological quality of included studies.

### 2.2 Eligibility criteria

Eligible criteria were cohort studies, in general population with no specific of age and gender that assess association of screen time with all sleep-related outcomes, which include sleep quality, sleep duration, night awakenings, sleep onset latency, bedtime daytime napping, sleep efficiency and insomnia. Screen time could be measured using subjective (e.g., self-reported) or objective (e.g. APP),. We excluded reviews, conference abstracts, editorials, commentaries, case reports, and studies that reported only unstandardized coefficients or lacked sufficient data for effect size conversion. There were no restrictions on publication language. Definitions of sleep outcomes are provided in **Supplementary Material 2**.

### 2.3 Search strategy

We systematically searched four databases (PubMed, EMBASE, PsycINFO, and Web of Science) to identify eligible studies published from inception until 06 March 2025 via predefined search terms, including “screen time”, “screentime”, “sleep quality”, “sleep time”, “sleep duration”, “Sleep onset latency”, “sleep Deprivation” and “cohort”. Details of the tailored search strategy in each database are shown in **Supplementary Material 1**. Search Strategy. No restriction was applied for publication time, and language, upon database searching.

### 2.4 Study selection

After removing duplicates using EndNote 20 software (Thomson Corporation, Stamford, USA), two investigators (H.X. and M.N.) independently screened the titles and abstracts of identified studies using Rayyan, an online literature management software (23). And then, two investigators independently assessed the full text of articles using predefined eligible criteria. Any discrepancies were resolved through discussion with the third investigator (P.B.), if required. To ensure consistency in screening, the investigators first calibrated their decisions by jointly reviewing a sample of records before full screening. Reasons for exclusion after full-text screening are detailed in **Supplementary Table 1**.

### 2.5 Data extraction

For each eligible study, the process of data extraction was independently performed by two reviewers (H.X. and M.N)using predesigned Microsoft Excel sheets. Any conflicts were resolved through discussion with the third investigator (P.B.), if required. We mainly extracted the following information: type of population, gender, first author, year of publication, study design, country, duration of follow-up, age, sample size, types of screen time, reported screen time duration in the study (e.g., TV watching time, mobile phone usage time, etc.), outcomes of interest, methods of outcome assessment, relevant effect size with 95% confidence intervals (CI), and confounding factors adjusted for.

### 2.6 Risk of bias

Two reviewers (H.X. and M.N.) independently performed risk of bias assessments of each included study using the Risk Of Bias In Non-randomized Studies of Exposures (ROBINS-E) tool (24).The results were visualized using the ROBVIS (25) tool. Any conflicts were resolved through discussion with the third investigator (P.B.), if required. The ROBINS-E tool assesses the risk of bias for the study outcome relevant to the systematic review question, which may not be the primary study outcome. It assesses risk of bias across seven domains; confounding, measurement of the exposure, participant selection, post-exposure interventions, missing data, measurement of the outcome, and selection of the reported result. The overall risk of bias for each study was determined using the ROBINS-E algorithm. Discrepancies were resolved by consensus discussion. Risk-of-bias assessments were subsequently incorporated into GRADE evaluation, with higher-risk studies leading to evidence downgrading and influencing credibility ceiling analyses.

### 2.7 Certainty of evidence

We used GRADE approach to assess the certainty of evidence for each sleep outcome and categorized the certainty of evidence as high, moderate, low, or very low (26). According to the GRADE standard, the certainty of evidence of cohort studies may start at low certainty and could be upgraded to moderate or high certainty if they present a dose–response gradient, a large effect, or if confounders likely minimize the effect. However, evidence certainty could also be downgraded because of serious limitations of the study, including indirectness, inconsistency, publication bias, or imprecision (27, 28).

### 2.8 Statistical analysis

All statistical analyses were performed using STATA 18.0 (StataCorp, College Station, TX, USA) (29). Outcomes were analyzed separately by type. For continuous outcomes (e.g. sleep duration), we used random-effects meta-analysis to pool β coefficients with 95% confidence intervals. Where necessary, effect sizes were converted to a common unit (minutes of sleep change per hour of screen time). For binary outcomes (e.g. short sleep), we pooled log-transformed odds ratios (log ORs) using a random-effects model. Risk ratios or hazard ratios were converted to odds ratios using established methods. To avoid metric inconsistency, we analyzed binary and continuous outcomes in separate models, and did not convert ORs into β coefficients.

Random-effects meta-analysis was conducted as the primary analysis to obtain pooled estimates, with heterogeneity assessed using the I² statistic and the between-study variance (τ²). Random-effects meta-regression with Knapp–Hartung adjustment was applied to account for between-study heterogeneity and to calculate 95% prediction intervals, estimating the likely range of effect sizes in future studies (30). Fixed-effect meta-analysis was performed as a sensitivity analysis to assess the robustness of results. Additionally, we conducted sensitivity analyses excluding studies with high risk of bias and those using objective sleep outcome measures to further examine the influence of study quality and measurement method on the overall estimates. Prespecified subgroup analyses were conducted when at least two studies were available, with tests of interaction used to evaluate whether subgroups differed significantly (P _interaction ≤_0.05). Subgroup factors included population type (infant, toddler, preschooler, child, adolescent, adult); age (>18 years vs. <18 years); risk of bias (high vs. low); country (China, US, UK, Australia, other), income (income, USD, 1,146–14,005 USD, >14,005 USD, according to World Bank thresholds); region (Asia, America, Oceania, Europe, other);follow-up time, dichotomized by outcome type: >2.7 years vs. ≤s.r years for continuous outcomes, and >2.5 years vs. ≤s.r years for binary outcomes; short sleep definition (aligned with the National Sleep Foundation (NSF)/the American Academy of Sleep Medicine(AASM) guidelines vs. not specified for binary outcome).Publication bias was assessed using Egger’s regression test and visual inspection of funnel plots, but only for meta-analyses including ten or more studies, as small-study bias tests are underpowered when k < 10.

## 3 Results

### 3.1 Characteristics of identified articles

**Figure 1:**
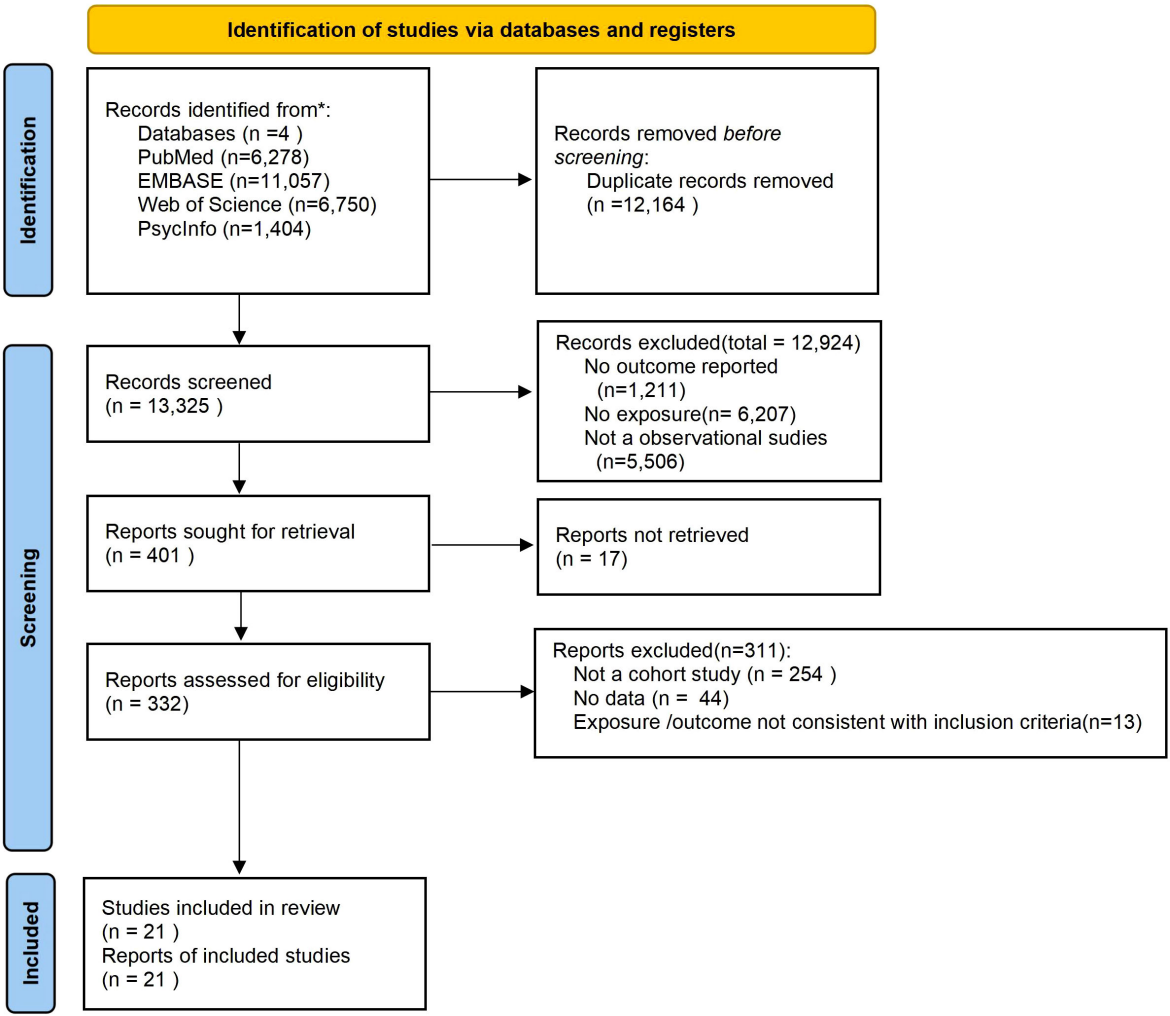
PRISMA flow diagram showing the identification, screening, eligibility assessment, and inclusion of studies in the systematic review.

A total of 25,489 studies were identified from database search, of which we excluded 13,325 duplicated studies. After titles, abstracts, and full texts screening, 21 studies (31–51) were included in the meta-analysis. The flow diagram of the selection is shown in **Figure 1**.

**Table 1** presents the detail baseline characteristic. This systematic review totally included 21 studies enrolled 548,338 participants. Of these studies, eight were conducted in Asia, two in Europe, six in America, three in Oceania, one in Brazil, one in Canada and one in Iran. 20 studies (31–50) examined the relationship between screen time and sleep duration across different life stages, including infants (n=2), toddlers (n=3), preschoolers (n=3), child (n=3), adolescents (n=6), and adults (n=4). And nine studies reported binary outcomes and 12 studies reported continuous outcomes. Three studies (31, 43, 50) have explored the relationship between screen time and insomnia. Among the participants, the median age was 12.76 years, the median female proportion was 41.5%, and the median length of follow-up was 3 years.

**Table 1:** Baseline characteristics of included studies.

Author, year	Country	Cohort, sample	Age, group	Screen time		Outcome	Covariates	Findings
				Types of exposure	Method of assessment			
Vijakkhana,2014 (31)	Thailand	/, 208	12.3 mo, Infant	all screen media	Parent reported use of all screen media	sleep duration	estimated media viewing, chronological age, gender, cosleeping status, maternal education in years, mother’s and father’s income	Evening screen time was negatively associated with sleep duration at 12 months (β = -0.14, 95% CI: -0.65 to 0.004); Bedroom screen time associated with sleep duration at 12 months (β = -0.05, 95% CI: -0.39 to 0.18).
Guo,2022 (32)	China HongKong	IGD, 41	22.3 ± 4.2y, Adult	Total screen time; mobile gaming time	Offscreen apps tracked total daily screen time; participants reported the mobile gaming time	sleep duration; early morning awakening; insomnia symptoms	sex, age, monthly household income, alcohol drinking, smartphone operating system, severity of problematic smartphone use, worry about social unrest in Hong Kong, and worry about being infected with COVID-19.	Total screen time was associated with shorter sleep duration(<7h) y(OR = 1.42,95%CI,0.97 to 2.03); Total screen time was associated with early morning awakening (OR = 0.94,95%CI,0.44 to 2.13);
Lan,2020 (33)	China HongKong	/, 2,903	3.9 (1.0)y, Preschooler	EMD use behaviors	Parent reported screen time on weekdays and weekends	sleep duration	age, parental education level, parental work status, housing area, family income, parental age, parental sleep duration and children out-door physical activity	Average screen time((>2.9 hours)was associated with short sleep duration (OR = 1.33,95%CI,0.98 to 1.81)
Chen,2020 (34)	Singapore	GUSTO, 552	5.5 (0.1)y, Preschooler	Total screen viewing time	Parent reported use of all screen time	sleep duration	the child’s sex, ethnicity, birth order, BMI at age 2 years, household income, and maternal education level, pre-pregnancy BMI and age at delivery, and study centre	Total screen time was associated with shorter sleep duration at age 5.5 y(OR = 0.99,95%CI,0.97 to 1.00)
Twenge,2017 (35)	US	MtF & YRBSS, 369,595	Adolescent	electronic device use time, TV viewing time	Participants reported the electronic device use time	sleep duration	sex, race, SES, and grade	Electronic device use was associated with shorter sleep duration (OR = 1.56,95%CI,1.50 to 1.62)
Emond,2021 (36)	US	Nurture, 452	12.3(0.8)m, Infant	Daily screen time	Parent reported use of daily screen time	sleep duration	infant age, sex and race; maternal age, marital/cohabitation status and education; and annual household income) and childcare characteristics (hours/week in relative-based or formal childcare, separately)	Higher levels of all screen time was associated with shorter sleep duration at age 12 mo(β=-1.6,95%CI,-5.9 to 2.7);
Nishioka,2022 (37)	Japen	JECS, 74,525	3y, Toddler	TV and DVD viewing time	Parent reported screen time	sleep duration	the parents’ highest level of education, co-residents, sleeping place, sleeping posture, and whether they slept at a fixed time	TV and DVD viewing time(>4h) was associated with sleep duration (OR = 1.03,95%CI,0.83,1.29),
Echevarria,2023 (38)	Brazil	2004 Pelotas Birth 1,851	15.7 (0.2)y, Adolescent	Total screen time	participants reported the total screen time	sleep duration	adolescent’s sex, skin color, maternal schooling, domestic agglomeration, whether the adolescent works, maternal depressive symptoms, sharing a bedroom, sharing a bed, alcohol consumption, smoking, and coffee consumption.	Average screen time((>9.0hours)was associated with sleep duration (β=-0.54,95%CI,-0.77 to -0.30),
Fogno,2022 (39)	France	ELFE & EPIPAGE2 5,700	8-9y, Child	Leisure screen time, School screen time	Parent reported use of all screen time	sleep duration	maternal education, household socio-professional category, family structure, single child, maternal age, household financial status, work status within the couple, dwelling with garden or yard, living area, dwelling occupancy index, gender, birth term, presence of a chronic or developmental pathology, indoors physical activity duration, SDQ, presence of infectious symptoms	Leisure screen time was associated with shorter sleep duration (β=-1.06,95%CI,1.02 to 1.10)
Yland,2015 (40)	US	FFCW 3,269	9y, Child	watching television per day; work on computer per day;	participants reported the screen time	sleep duration	sex, race/ethnicity, parental relationship, and mother’s education	Work on computer time was associated with weekday sleep duration (β= -0.05),
Yan,2022 (41)	China	/, 734	54m, Toddler	screen time	participants reported the screen time	sleep duration	Age, gender, mother’s age at birth, mother’s education level, father’s education level, father’s smoking status, family’s per capita monthly income, whether it is a premature birth, birth weight, monthly age multiplied by outdoor activity time, monthly age multiplied by screen time.	Screen time was associated with sleep duration (β=0.03,95%CI,-0.20 to 0.26)
Nevarez,2010 (42)	US	Viva, 1,802	2y, Toddler	TV viewing time	Parent reported use of tv/video viewing time	sleep duration	age, prepregnancy weight, and their height, from which we calculated prepregnancy body mass index, parity, marital status, educational attainment, foreign-born status, and annual household income. After delivery, mothers reported their infants’ gender and race/ ethnicity and the total number of household members.	TV/video viewing time (>4h) was associated with sleep duration(β=-0.13,95%CI,-0.19 to 0.08)
Li,2021 (43)	US	Add Health 9,279	16.06 (0.02)y, Adolescent	sedentary screen time	participants reported the screen time	sleep duration	demographic variables, socioeconomic status (SES), health behaviors, and sleep duration at Wave I, and SES, health, behaviors, MVPA, and SST at Wave V. Demographic characteristics included age at Wave I, biological sex, race/ethnicity, and nationality. Among the Wave I (Mage = 16) covariates, SES included household in come, highest education of either of the parents, and neighborhood safety. Health behaviors included smoking in the past 30 days, drinking in the past 12 months, body mass index, self-rated health, and physical limitation.	Sedentary screen time(>22) was associated with shorter sleep duration(RR = 1.19,95%CI,1.06 to 1.80),
Liu,2019 (44)	China	/, 4,733	18.3 (1.7)y Adolescent	Mobile phone use	participants reported the mobile phone use time	sleep duration; Insomnia	age, sex, lifestyle practice and health condition	Mobile phone use time was associated with shorter sleep duration (OR = 1.26,95%CI,-0.81to 1.96); Mobile phone use time was associated with higher Insomnia risk (OR = 1.28,95%CI,0.99to 1.67)
Tavernier,2014 (45)	Canada	/, 942	19.02(0.90)y, Adult	Media use time	Self-reported	sleep duration	age, gender and parental education	Media use time was associated with sleep duration (β=-0.03,95%CI,-0.27to 0.18)
Magee,2014 (46)	Australian	Cohort K, 3,427	5-6y, Preschooler	TV viewing time, total media use, Computer Use	Parent reported screen time	sleep duration	child’s sex and baseline obesity status, with sleep problems, household income, and maternal education	Total media use time was associated with sleep duration (β=-0.03,95%CI,-0.5to -0.01)
Rosa2024 (47)	New Zealand	NZAVS, 21,936	51.9(13.6)y, Adult	Total amount of screen-based leisure time	participants reported the total screen time	sleep duration	demographic indicators, weekly time in other activities, personal- ity, beliefs	Total screen-based leisure time was associated with shorter sleep duration (β=-0.03,95%CI,-0.5to -0.01)
Sampasa-Kanyinga2022 (48)	UK	UK Biobank 31,361	56.1(7.5)y, Adult	Daily DST	participants reported the DST	sleep patterns	age, sex, follow-up time, BMI, socioeconomic status, physical activity level, vegetable and fruit intake, shift work, cigarette smoking, alcohol consumption, mental health issues, and history of major cardiovascular disease and cancer.	High follow-up DST was associated with shorter sleep duration(OR = 1.13,95%CI,1.04 to 1.23)
Nagata2024 (49)	US	ABCD, 11,875	12.02(0.66)y Adolescent	Overall screen usage	participants reported total recreational screen time	sleep disturbance; sleep duration	age, sex, race/ethnicity, household income, parent education, adverse childhood experiences, depression symptoms, melatonin use, study site, and the respective sleep variable at Year 2.	Total recreational screen Time associated with total sleep disturbance(OR = 1.02,95%CI,1.01 to 1.04) Total recreational screen Time associated with total sleep duration(β=-0.03,95%CI,–0.04 to -0.02)
Nikooharf Salehi2025 (50)	Australia	LSAC, 2,064	10-12y, Child	Screen time of 24-h period	participants use time-use diaries (TUDs) record	Sleep duration	age, gender, BMI, socioeconomic position (SEP), sibling size, school attendance on the day of TUD completion (yes/no), whether the TUD was completed on a weekday or weekend, physical activity timing, and presence of medical conditions	screen time was inversely associated with sleep duration(β=-0.071,95%CI,-0.114 to -0.0115)
Lin,2021 (51)	China	/, 1089	13-19y, Adolescent	TV and DVD viewing time	Parent reported screen time	Insomnia	age, gender, and father’s education	Social media time was associated with High of insomnia symptoms risk(β=0.49)

Of the 21 studies, five did not specify the names of the cohort studies, while the remaining 16 studies came from different cohorts, with no overlap in the surveyed populations.

### 3.2 Risk of bias assessment

All studies were observational in design, and so risk of bias assessments were performed using the ROBINS-E tool(**Supplementary Figures 8, 9**). Across the included studies, several consistent sources of potential bias were identified, particularly in the domains of exposure measurement, outcome assessment, and participant selection. In Domain 2 (Risk of bias arising from measurement of the exposure), nearly all studies were judged to raise some concerns, as screen time was predominantly assessed using subjective methods such as self-reports or parent-reports. These approaches are inherently prone to recall and reporting errors, which may result in exposure misclassification and introduce bias. Similarly, in Domain 6 (Risk of bias arising from measurement of outcomes), most studies were rated as having some concerns, given that sleep outcomes were largely assessed using subjective instruments, with only a single study employing an objective measurement tool. This raises the possibility that outcome assessors’ knowledge of participants’ exposure could have influenced their evaluations, thereby increasing the risk of bias in outcome measurement.

Regarding Domain 3 (Risk of bias in selection of participants into the study), several studies initiated follow-up from infancy, recording exposure from its onset, and were therefore considered at low risk of bias. In contrast, studies that did not commence follow-up from birth may have missed participants who developed early sleep problems potentially related to screen exposure, and were consequently rated as having some concerns in this domain.

Overall, these assessments highlight that while the majority of studies were of acceptable quality, particular caution should be exercised in interpreting findings related to subjective exposure and outcome measurement.

### 3.3 Screen time and sleep duration

Among the 20 cohort studies (31–50) (547,249 participants) examining the association between screen time and sleep duration, 11 studies (31, 36, 38, 40–42, 45–47, 49, 50) (48,560 participants) reported continuous sleep outcomes, which were standardized to a 1-hour increase in daily screen time for comparability. A conventional random-effects meta-analysis of these 11 studies indicated that longer screen time was associated with shorter sleep duration (β = −0.05; 95% CI: −0.08 to −0.03)(**Table 2**). To further address between-study heterogeneity, a random-effects meta-regression with Knapp–Hartung adjustment was performed, which indicated moderate heterogeneity (I² = 61.3%, τ² = 0.00045) and a 95% prediction interval ranging from −0.092 to −0.009, entirely within negative values (**Table 3**).

**Table 2:** Summary findings of screentime and sleep outcome.

Outcome	Studies, n	Effect size (95%CI)	GRADE assessment	*P-* interaction	I- square, %
Continuous Outcome					
Sleep duration	11	-0.05(-0.08 to -0.03)	Very Low	/	61.3%
Insomnia	2	0.41(0.18 to 0.63)	Low	/	69.3%
Binary Outcome					
Sleep duration	9	1.25(1.08 to 1.41)	Moderate	/	96.5%
Subgroup (Continuous Outcome)					
Country					
US	4	-0.03(-0.05 to -0.02)			7.0%
Australia	2	-0.07(-0.11 to 0.04)			0.0%
Other countries	5	-0.12(-0.29 to 0.05)			78.0%
Overall	11		Low	0.81	
Region					
Asia	2	-0.02(-0.21 to 0.16)			0.0%
America	4	-0.03(-0.05 to 0,02)			7.0%
Oceania	3	-0.05(-0.08 to -0.02)			48.0%
Other region	2	-0.25(-0.74 to 0.24)			89.8%
Overall	11		Low	0.33	
Age					
<18 years old	9	-0.07(-0.11 to -0.03)			68.7%
≥18 years old	2	-0.03 (-0.05 to -0.01)			0.0%
Overall	11		Low	0.48	
Type of Population					
Infant	2	-0.04(-0.11 to 0.04)			0.0%
Toddler	2	-0.08(-0.14 to -0.02)			0.8%
Preschooler	1	-0.07(-0.12 to -0.02)			
Child	2	-0.07(-0.12 to -0.02)			0.0%
Adolescent	2	-0.25(-0.71 to 0.21)			94.3%
Adult	2	-0.03(-0.05 to -0.01)			0.0%
Overall	11		Low	0.31	
Follow-up time					
< 2.5 years	5	-0.03(-0.04 to -0.02)			0.0%
≥ 2.5 years	6	-0.07(-0.13 to -0.01)			76.3%
Overall	20		Low	0.72	
Subgroup (Binary Outcome)					
Country					
US	2	0.32(0.05 to 0.558)			94.3%
China	3	0.47(0.28 to 0.67)			9.2%
Other countries	4	0.07 (0.04to 0.10)			9.9%
Overall	9		Low	0.004	
Region					
Asia	4	0.35(0.05 to 0.64)			83.4%
America	2	0.32(0.05 to 0.58)			94.3%
Oceania	1	-0.03(-0.26 0.20)			
Europe	2	0.08(0.02 to 0.14)			50.1%
Overall	9		Low	0.099	
Age					
<18 years old	6	0.23(0.08 to 0.31)			98.2%
≥8. years old	3	0.23 (0.04 to 0.41)			47.5%
Overall	9		Low	0.81	
Type of Population					
Preschooler	2	0.66(0.35 to 0.96)			92.4%
Adolescent	3	0.33(0.12 to 0.54)			88.6%
Adult	2	0.16(-0.01 to 0.33)			228.5%
Other population	2	0.06(0.03 to 0.09)			0.0%
Overall	9		Low	0.21	
Follow-up time					
< 2.7 years	5	0.27(0.09 to 0.45)			83.3%
≥3.3 years	4	0.17(-0.07 to 0.40)			98.1%
Overall	9		Low	0.46	
Short sleep definition					
Aligned with NSF/AASM guidelines	4	0.37(0.14 to 0.56)			67.6%
Not specified	5	0.15(-0.04 to 0.33)			98.5%
Overall	9		Low	0.43	

**Table 3:** Summary of 95% prediction intervals from random-effects meta-regression analyses.

Outcome measure	95% Prediction Interval	I² (%)	τ²
Sleep duration (continuous outcomes only)	(–0.092 to –0.009)	61.3	0.00045
Inadequate sleep duration (binary outcomes)	(0.875 to 1.790)	97.2	0.033

Prediction intervals represent the expected range for the true effect size in a future study. I² indicates the proportion of total variability due to between-study heterogeneity, and τ² denotes the estimated between-study variance.

The remaining nine cohort studies (32–35, 37, 39, 43, 44, 48), including 498,689 participants, reported binary outcomes of short sleep. A conventional random-effects meta-analysis indicated that greater screen time was associated with a higher risk of short sleep (OR = 1.25; 95% CI: 1.08 to 1.40) (**Table 2**). To further account for between-study heterogeneity, a random-effects meta-regression with Knapp–Hartung adjustment was conducted across these nine studies. To further address between-study heterogeneity, we performed a random-effects meta-regression with Knapp–Hartung adjustment. The analysis revealed high heterogeneity (I² = 97.2%, τ² = 0.033), with a 95% prediction interval ranging from 0.87 to 1.79, encompassing the null value (OR = 1) (**Table 3**).

We conducted subgroup and corresponding meta-regression analyses by country, region, age group, population type, and follow-up duration (**Supplementary Table 2** and **Table 3**). According to WHO classification (52), only one study was conducted in a middle-income country, while the remaining studies were from high-income settings; therefore, income-based subgroup analysis was not performed. For continuous outcomes, there were no significant differences in the association between screen time and reduced sleep duration across countries (P _interaction_=0.81) or regions (P _interaction_=0.33). The association also did not differ significantly by age group (rou vs. <18 years; P _interaction_=0.48, population type (P _interaction_=0.31), or follow-up duration, which was dichotomized at the median value of 2.7 years (P _interaction_=0.72). Subgroup analysis by risk of bias was not conducted, as all included studies were assessed as having moderate risk. For binary outcomes, subgroup and meta-regression analyses were similarly conducted. A significant difference was observed by country (P _interaction_= 0.004), suggesting that the association between screen time and the odds of short sleep varied across countries. However, no significant effect modification was found across regions (P _interaction_= 0.099), age groups (P _interaction_= 0.81), population types (P _interaction_= 0.21), short sleep definition (P _interaction_= 0.43) or follow-up duration, which was dichotomized at the median value of 2.5 years (P _interaction_= 0.46)(**Table 2**). Subgroup analysis by risk of bias was not performed, as only one study was rated as high risk, while the remaining studies were assessed as having moderate risk. Details of short sleep definitions are provided in the **Supplementary Material 2**.

Sensitivity analyses were conducted for binary outcomes, as high-risk studies and those using objective outcome measures were both specific to this outcome type. Including 9 studies yielded a pooled effect size of OR = 1.25 (95% CI: 1.08 to 1.41; *p* < 0.001) with high heterogeneity (*I²* = 96.5%) (**Supplementary Figure 1**).After excluding studies at high risk of bias (n = 8), the pooled effect size decreased to 1.15 (95% CI: 1.05 to 1.18; p < 0.05) (**Supplementary Figure 6**), and heterogeneity was markedly reduced (I² = 61.4%). In contrast, excluding studies that employed objective outcome measures (n = 8) produced a pooled effect size of 1.29 (95% CI: 1.07 to 1.5; p < 0.001) (**Supplementary Figure 7**), which was highly consistent with the original estimate, while heterogeneity remained extremely high (I² = 96.9%). Moreover, results from the fixed-effect model were comparable to those from the random-effects model, supporting the robustness of the findings. (**Supplementary Figures 1, 2**) For continuous outcomes, results from the random-effect model (β = −0.05; 95% CI: −0.08 to −0.03) were highly consistent with those from the fixed-effects model (β = −0.03; 95% CI: −0.04 to −0.03), supporting the robustness of the findings despite potential between-study heterogeneity. (**Supplementary Figures 3, 4**).

For continuous outcomes, the funnel plot appeared largely symmetrical, and Egger’s test showed no significant evidence of publication bias (P = 0.08) (**Supplementary Figure 5**). For binary outcomes, Egger’s test was not conducted due to the small number of included studies (n = 9), as tests for funnel plot asymmetry are not reliable when fewer than 10 studies are available.

### 3.4 Screen time and insomnia

Three studies (32, 44, 51) involving 5,872 participants investigated the relationship between screen time and insomnia. Two of these studies (44, 51), both using the Insomnia Severity Index (ISI), conducted quantitative analyses and found limited evidence of a significant positive association between screen time and an increased risk of insomnia (β = 0.41, 95% CI, 0.18 to 0.63) (**Table 2**). The results of the fixed-effect model (**Supplementary Figures 12, 13**) and random-effect model were relatively consistent. The cohort study by Guo (32) revealed that longer total screen time was associated with greater reported severity of insomnia symptoms (OR = 0.79; 95% CI, 0.51 to 1.14). Notably, this study did not use the ISI to assess insomnia symptoms. Instead, it introduced new measurement variables, including difficulty initiating sleep, difficulty maintaining sleep, and early morning awakening. Participants reporting any of these symptoms were categorized as having “any insomnia symptoms.” In summary, increased screen time is linked to more severe insomnia symptoms.

### 3.5 Other sleep outcomes

Guo (32) demonstrated that longer mobile gaming time was associated with a threefold increase in the risk of difficulty initiating sleep (OR = 3.05, 95% CI: 1.51 to 6.24), and 2.2 times greater risk of difficulty maintaining sleep (OR = 2.19, 95% CI: 1.18 to 3.74). Fogno (39) found a positive association between both total screen time and leisure screen time with the onset or worsening of sleep difficulties (OR = 1.01, 95% CI: 0.95 to 1.06).

Lan (33) indicated that every additional hour of non-portable electronic device use was associated with a 3-minute delay in social jetlag for boys. In contrast, Emond (36) found no significant association between screen time and daytime sleep or nighttime awakenings.

Nishioka (37) revealed that increased usage of TV/DVDs and portable electronic devices (PEDs) was linked to a higher risk of late bedtimes. Yan (41) reported that for children aged 36 to 54 months, screen time was associated with delayed bedtimes (β = 0.22, 95% CI: 0.05 to 0.39). (Delayed bedtimes to the actual clock time when an individual goes to sleep whereas eveningness is a broader chronotype trait reflecting a preference for later activity and alertness during the day and night (53). Specifically, for every additional hour of screen time, bedtime was delayed by 13.2 minutes.

## 4 Discussion

### 4.1 Main findings

To our knowledge, this is the first systematic review to explore the association between screen time and sleep outcomes across diverse populations, exclusively including cohort studies. This meta-analysis systematically evaluated the relationship between screen time and sleep outcomes across different populations, including infants, toddlers, preschoolers, children, adolescents, and adults. Our findings reveal consistent and significant adverse associations between increased screen time and poorer sleep outcomes across these age groups.

The primary findings were twofold: firstly, that longer screen time was positively correlated with shorter sleep duration, irrespective of age; and secondly, that prolonged screen time increases the risk of insomnia. Furthermore, although a meta-analysis was not conducted for other sleep-related outcomes, a qualitative analysis indicates that longer screen time can precipitate numerous sleep problems, including a tendency to go to bed later and difficulty falling asleep.

### 4.2 Subgroup and sensitivity analyses

Our subgroup and sensitivity analyses provided complementary insights into the robustness and potential sources of heterogeneity in the association between screen time and sleep outcomes. Subgroup analyses were conducted separately for binary and continuous outcomes. For most subgroup factors, including age, region, population type, short sleep definition and follow-up duration, no statistically significant interactions were observed (P _interaction_ > 0.05). However, in the binary outcome analysis, a significant interaction by country was observed (P _interaction_ = 0.004), suggesting that national context may influence the association between screen time and short sleep. Given the limited number of studies in several subgroups, especially within the binary outcome analysis, these analyses may have been underpowered to detect modest effect modifications. Therefore, non-significant findings should be interpreted with caution. Sensitivity analyses showed that excluding studies at high risk of bias reduced both the pooled effect size and heterogeneity, indicating that lower-quality studies may have slightly inflated the overall estimate. In contrast, excluding studies employing objective sleep measures did not materially alter the results, suggesting that measurement type was unlikely to be a major contributor to between-study variability. Taken together, these results suggest that the association between screen time and sleep outcomes is generally consistent across populations and study designs. However, the magnitude of the effect should be interpreted cautiously, in light of residual heterogeneity and the limited number of studies within certain subgroups.

### 4.3 Comparison with previous studies

A substantial body of prior research exists concerning the correlation between screen time and sedentary behavior, including the influence of other risk factors.

In recent years, studies (54) have been conducted targeting adolescents, which have yielded significant correlations between screen time and the risk of myopia. Additionally, an examination of the extant literature on the association between screen use and ASD has revealed that the existing evidence does not fully support the claimed association (55). Furthermore, there has been considerable attention devoted to the study of screen time and risk factors such as high blood pressure (56), low back pain (57), metabolic syndrome (58, 59), and obesity (60) in adolescents. The increasing focus on screen time among adolescents in recent years is evident.

Prior research on the correlation between screen time and sleep outcomes has been conducted on individuals below the age of 18 (15–19). In this study, in comparison to previous studies, the population is defined as all individuals, without any restrictions. Secondly, with regard to the type of study, only cohort studies were selected, which allows for a more robust demonstration of the association between screen time and sleep duration. Furthermore, additional subgroup analyses were conducted to elucidate the source of heterogeneity.

### 4.4 Strengths and limitations

The present study contributes to the growing body of evidence supporting the association between screen time and sleep duration. Our findings indicate a negative association between screen time and sleep duration across both binary and continuous outcomes. However, the strength of this association varied across populations, and no significant modification was observed by age or cultural factors.

Despite a considerable number of studies investigating this relationship, several limitations remain. First, although a comprehensive search strategy was applied, relevant studies may still have been inadvertently omitted. The search was not linked to Medical Subject Headings (MeSH), which may have limited the inclusion of studies addressing various forms of electronic media.

Second, the number of included studies in certain subgroups was limited, which may have reduced the statistical power to detect effect modification. Additionally, for subgroups with fewer than ten studies, formal publication bias testing such as Egger’s test was not conducted, in accordance with methodological recommendations.

Third, a formal dose-response meta-analysis was not performed due to the lack of exposure-response data with multiple quantitative screen-time categories. Furthermore, the categorization of both screen time and sleep outcomes varied substantially across studies, limiting the feasibility of modeling exposure gradients. Future studies with harmonized exposure definitions and multiple screen-time levels are warranted to allow dose-response assessment.

Fourthly, the analysis was restricted to β coefficients and odds ratios, focusing only on sleep duration and insomnia. Notably, only three studies addressed insomnia risk, precluding quantitative synthesis for this outcome. Finally, outcome definitions were heterogeneous. For example, symptoms such as difficulty initiating or maintaining sleep were treated as “other sleep outcomes” rather than formal insomnia diagnoses, as most studies reported them based on self-report rather than clinical criteria. This variability may have introduced additional heterogeneity and limited direct comparability across studies.

### 4.5 Implications

The findings of this meta-analysis underscore a significant association between screen time and sleep outcomes, with important implications for public health, clinical practice, and future research. The observed negative association between increased screen time and reduced sleep duration, along with a heightened risk of insomnia, highlights the need for effective interventions to reduce screen exposure, particularly among individuals at risk of sleep disturbances.

While screen time appears to be a modifiable factor influencing sleep, these findings should be interpreted with caution due to the observational nature of the included studies, the potential for bidirectional relationships, and the small-to-moderate effect sizes with uncertain clinical relevance. Public health and clinical efforts may benefit from low-risk, context-sensitive strategies such as promoting good sleep hygiene and reducing screen use before bedtime, rather than applying rigid and universal screen-time thresholds. Health education initiatives that raise awareness of the potential impacts of screen use on sleep, combined with practical guidance on timing, content, and device use, may also prove effective. Integrating screen-time monitoring and sleep-hygiene education into school and community programs could foster healthier behavioral patterns and improved sleep outcomes.

Although this study adds to the growing body of evidence, several gaps remain. Further longitudinal and experimental research is essential to clarify the causal direction of the screen time–sleep relationship. Such studies could deepen our understanding of the underlying mechanisms and inform the development of more precise, evidence-based recommendations tailored to different populations.

In addition, future studies should aim to conduct formal dose–response analyses, which were not feasible in the present meta-analysis due to the lack of consistent exposure categorizations and quantitative data across studies. Clarifying whether a threshold effect or linear association exists would offer valuable insights for practical guidelines.

Expanding the scope of future research to include additional sleep outcomes such as sleep quality, sleep latency, and sleep disorders would allow for a more comprehensive evaluation of the broader impact of screen exposure on sleep health. In light of the wide variation in screen-use behaviors and sleep norms across cultural and environmental contexts, future studies should also explore the potential moderating role of sociocultural factors to ensure that recommendations are both contextually relevant and globally applicable.

## 5 Conclusion

In summary, our systematic review and meta-analysis confirms the positive effect of screen time on sleep duration, with adolescents’ screen time use being particularly influential. This study highlights the need for public health initiatives to raise awareness of these effects, especially among adolescents. Further research, including experimental and longitudinal studies, is necessary to delve deeper into the complex relationship between screen time use and sleep duration, while taking into account potential moderating factors such as cultural differences.

## Data Availability

The original contributions presented in the study are included in the article/**Supplementary Material**. Further inquiries can be directed to the corresponding author.
