# Supplementary material for: The association of screen time and the risk of sleep outcomes: a systematic review and meta-analysis
Source: Front Psychiatry. 2025 Dec 17;16:1640263. doi: 10.3389/fpsyt.2025.1640263 (PMC12754674; doi:10.3389/fpsyt.2025.1640263)
Supplement: Supplementary file 1 [file DataSheet1.zip › Supplementary Figures.docx]

**Supplementary Figures**


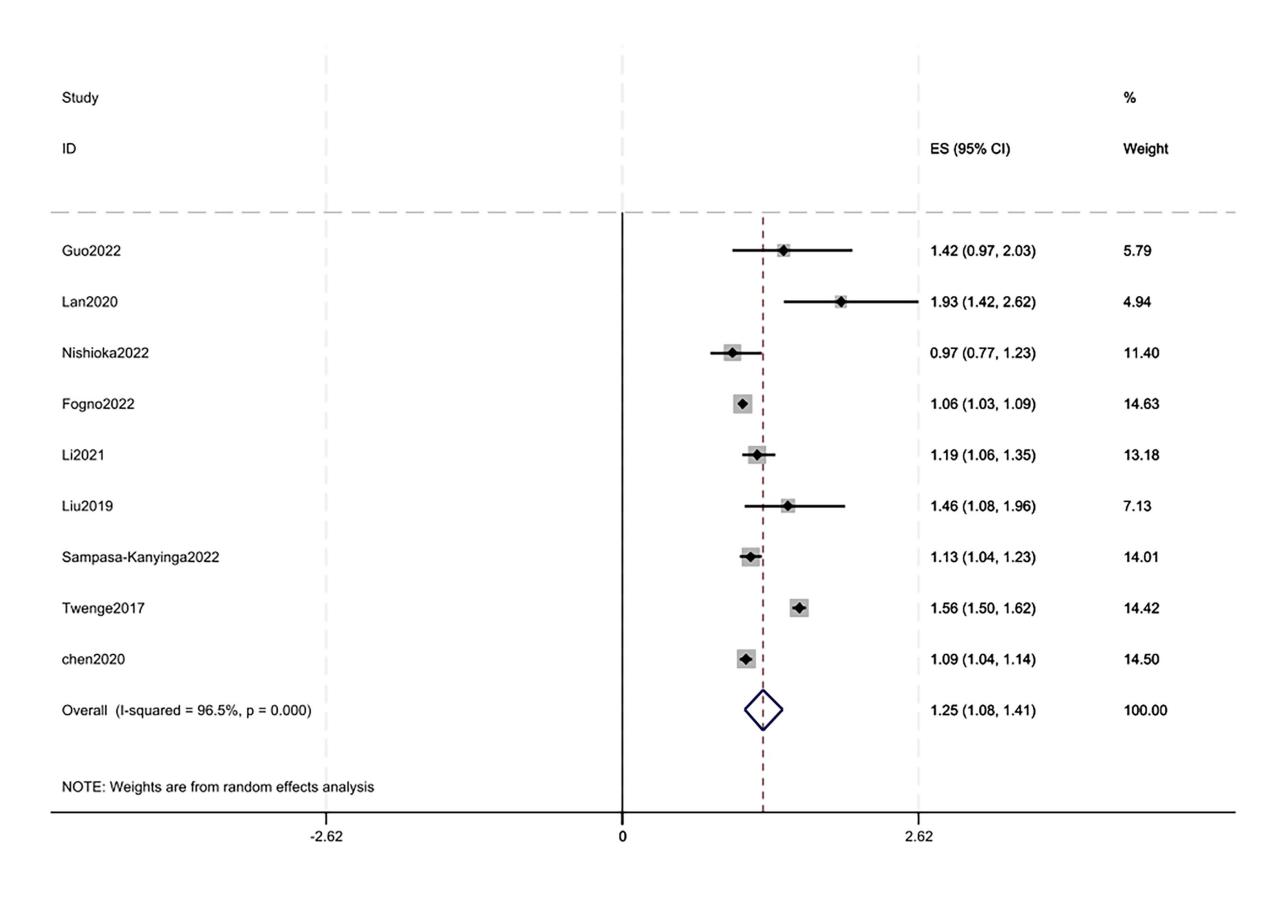


**Supplementary Figure 1.** Meta-analysis of binary outcomes: screen time and sleep duration(Random-effects model)

**
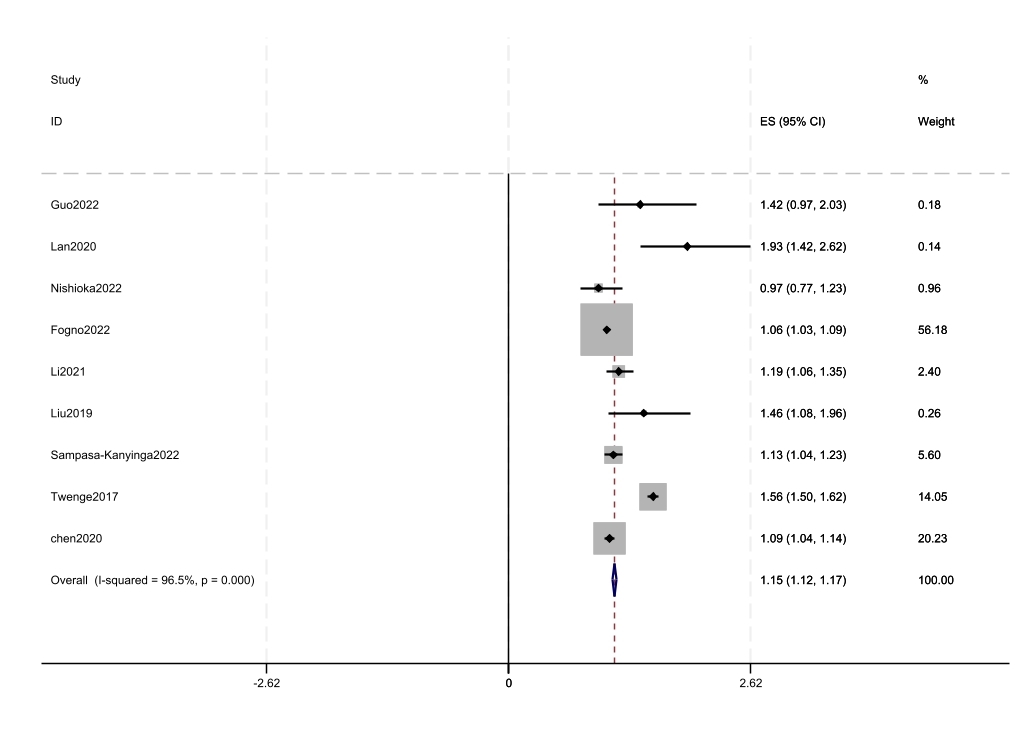
**

**Supplementary Figure 2.** Meta-analysis of binary outcomes: screen time and sleep duration (Fixed-effects model)

**
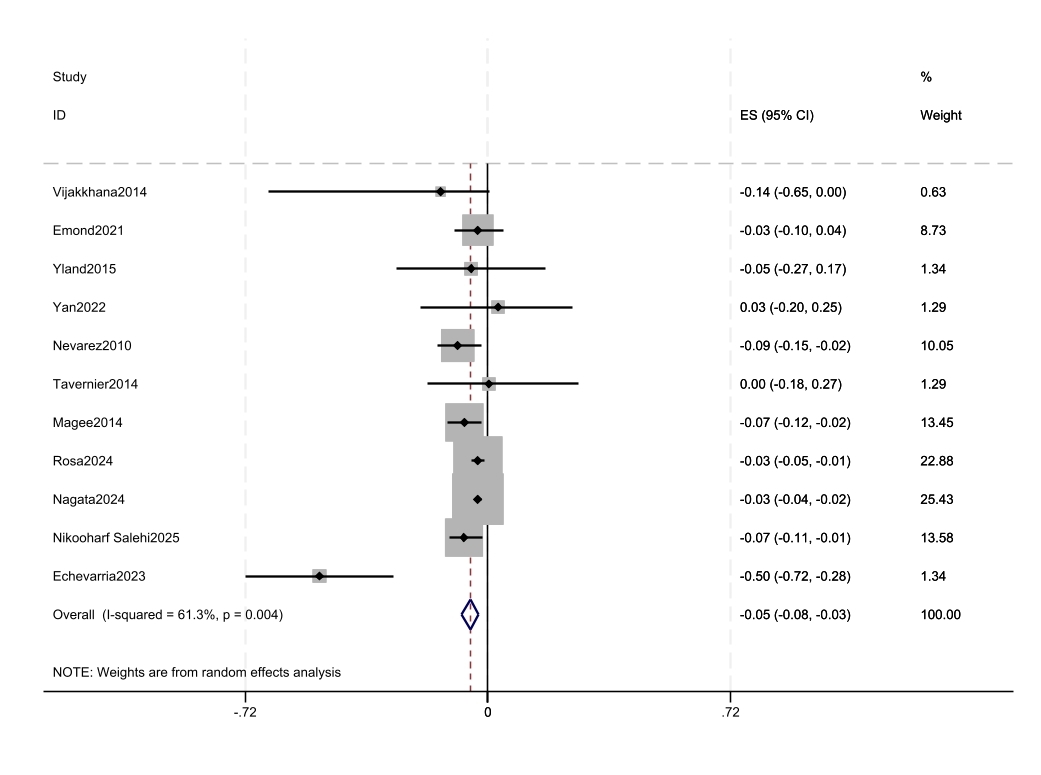
**

**Supplementary Figure 3.** Meta-analysis of continuous outcomes: screen time and sleep duration(Random-effects model)

**
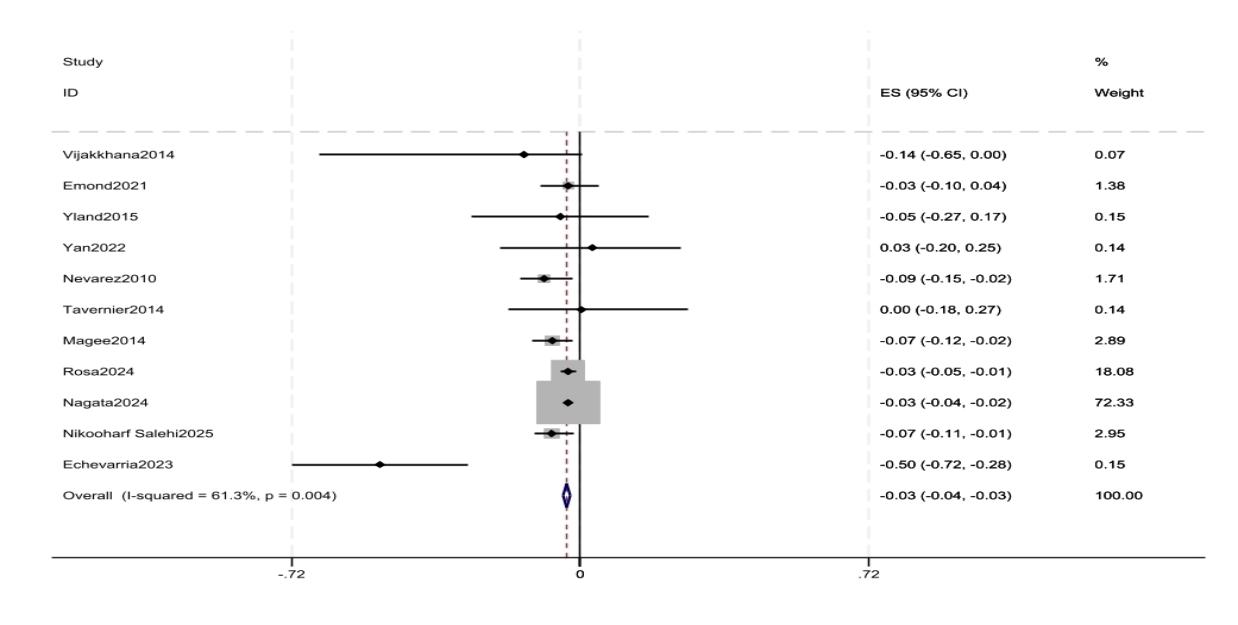
**

**Supplementary Figure 4.** Meta-analysis of continuous outcomes: screen time and sleep duration(Fixed-effects model)

**
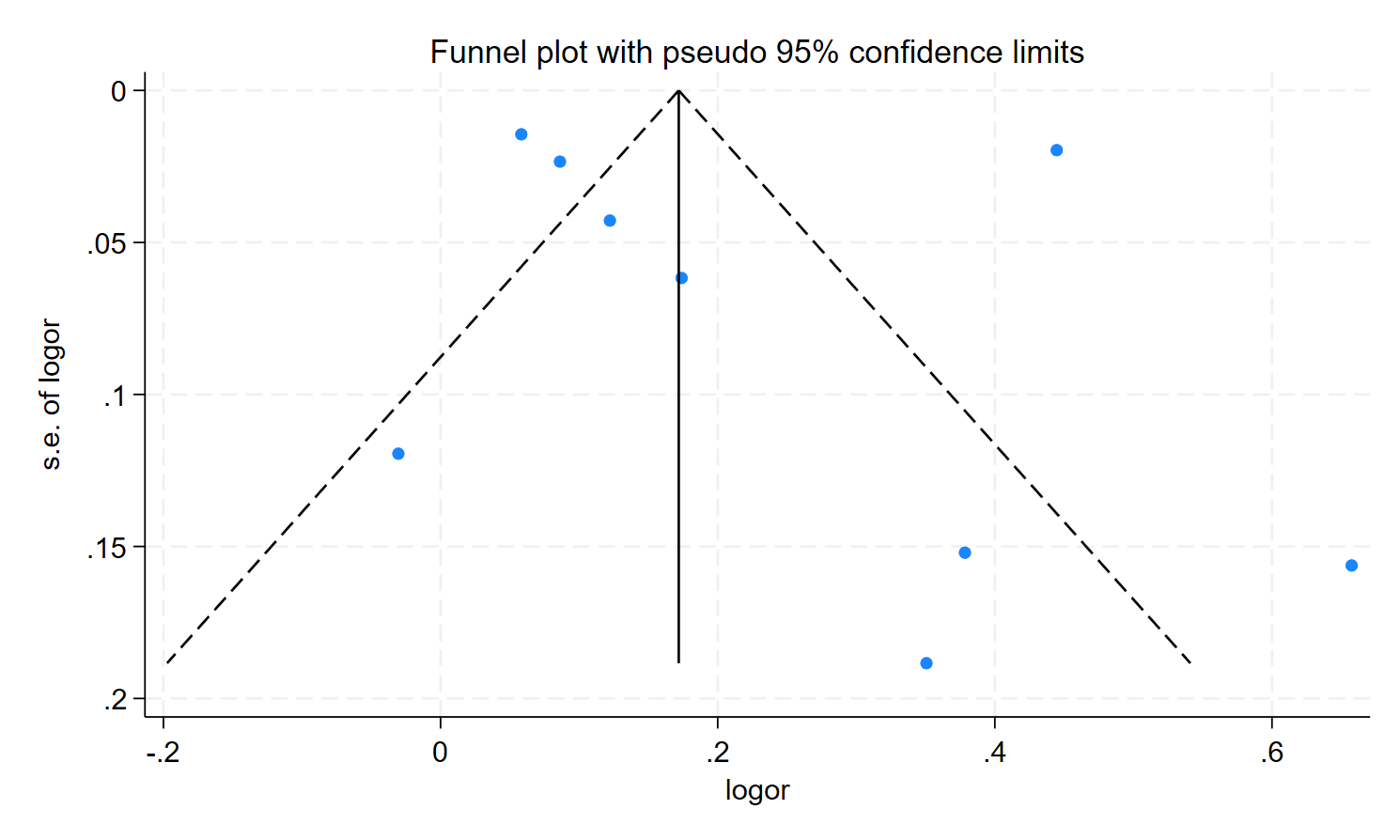
**

**Supplementary Figure 5.** Funnel plot for continuous outcomes

**
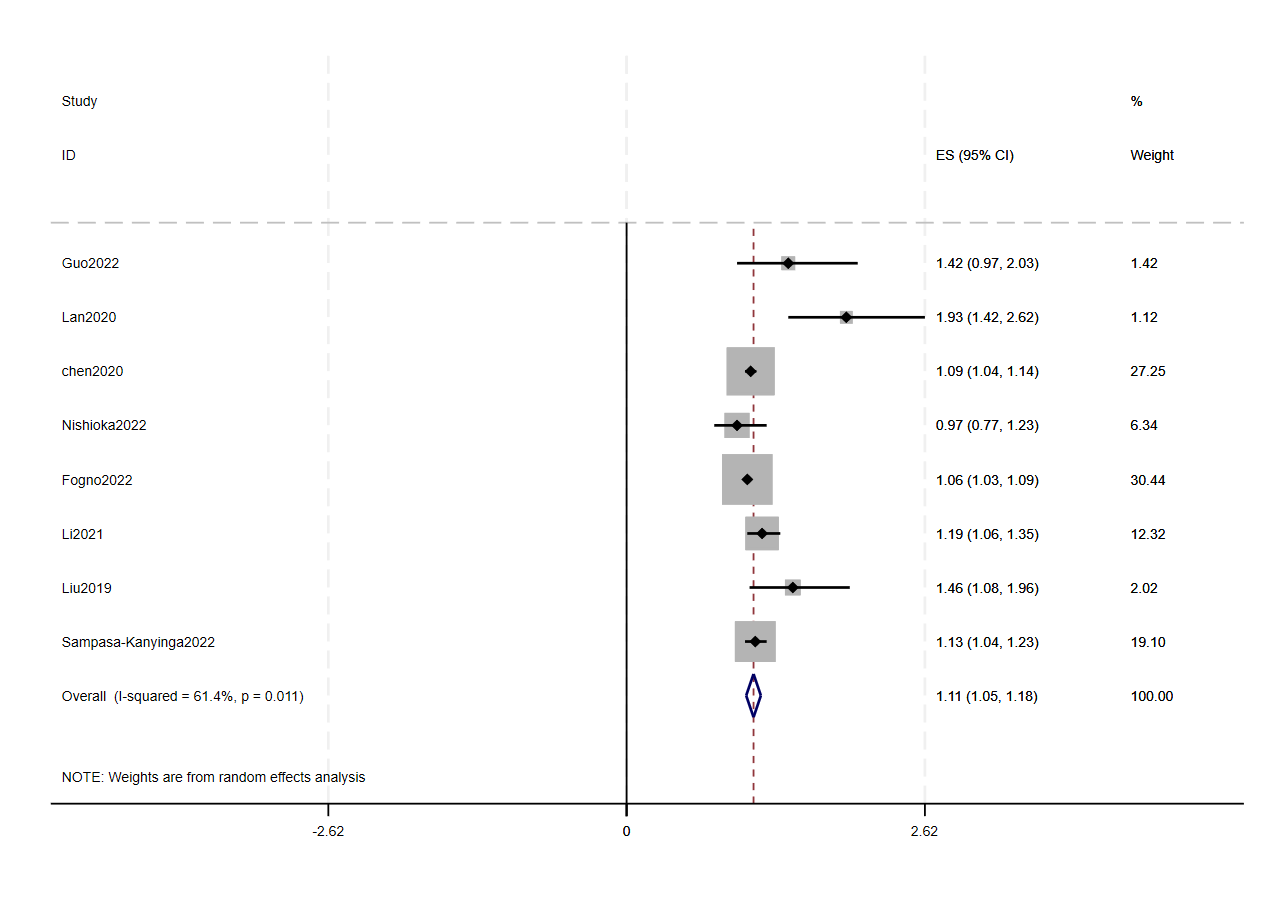
**

**Supplementary Figure 6.** Forest plot of the sensitivity analysis excluding studies at high risk of bias.

**
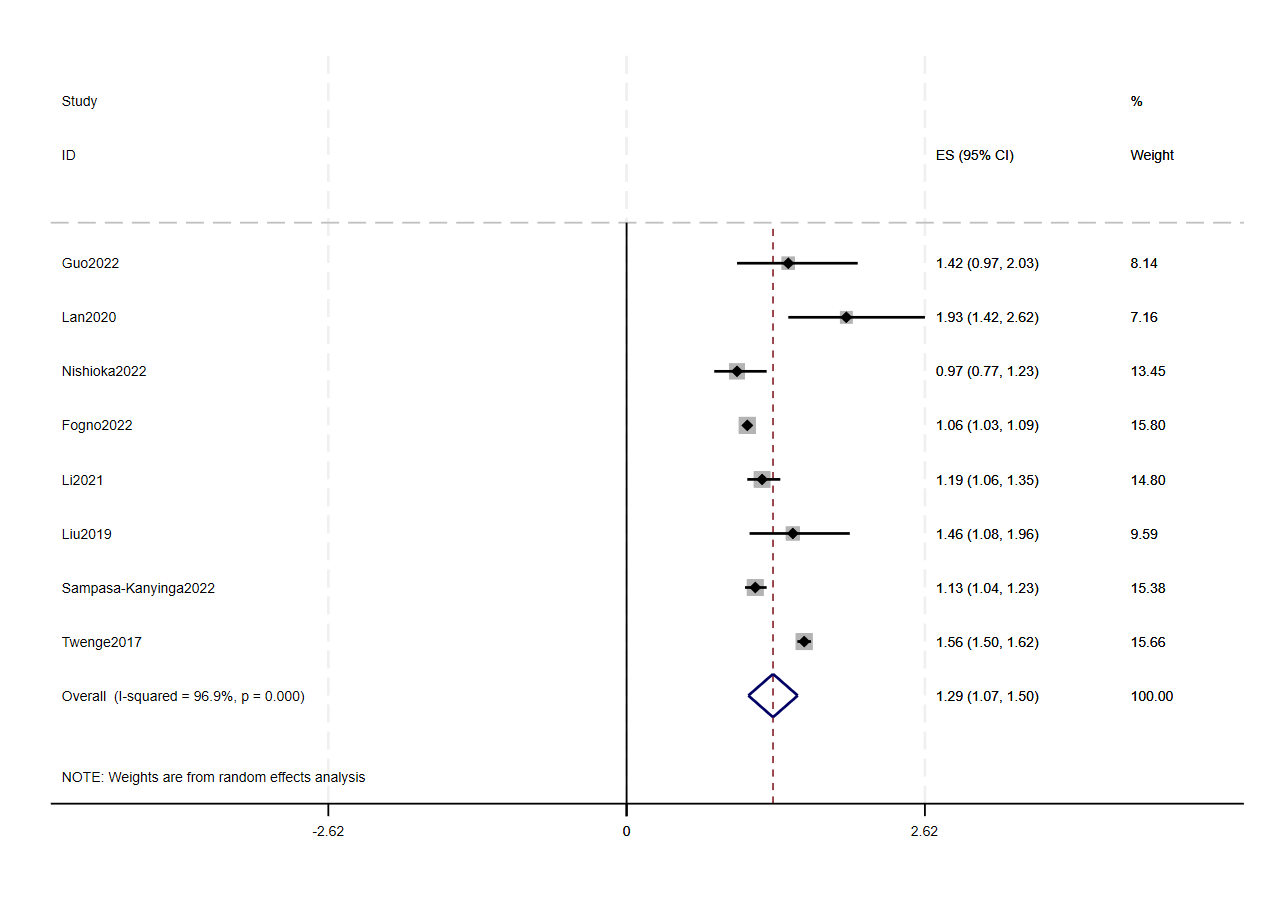
**

**Supplementary Figure 7.** Forest plot of sensitivity analysis excluding studies using objective outcome measures.


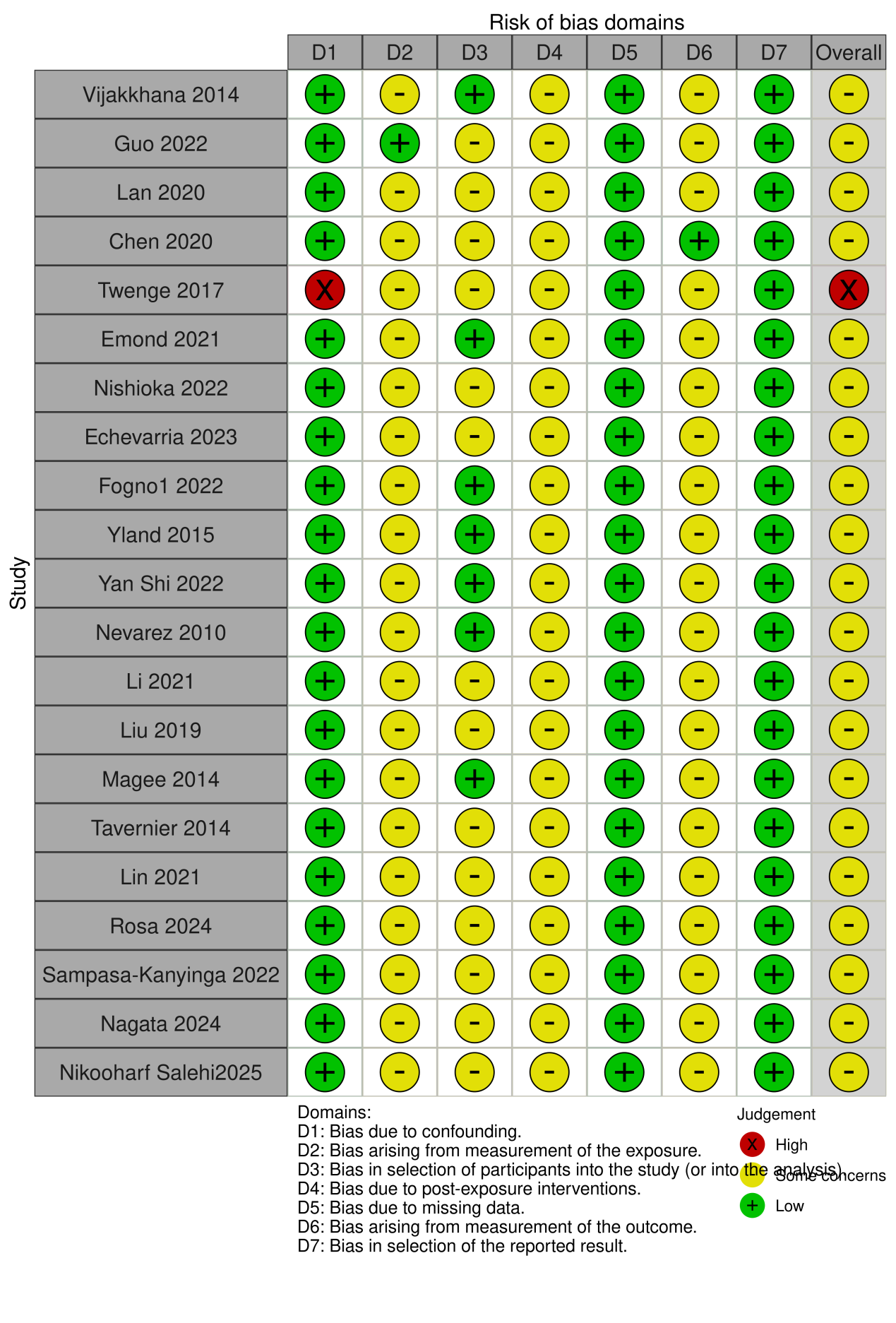


**Supplementary Figure 8.** Traffic light plot of risk of bias assessment for included cohort studies.


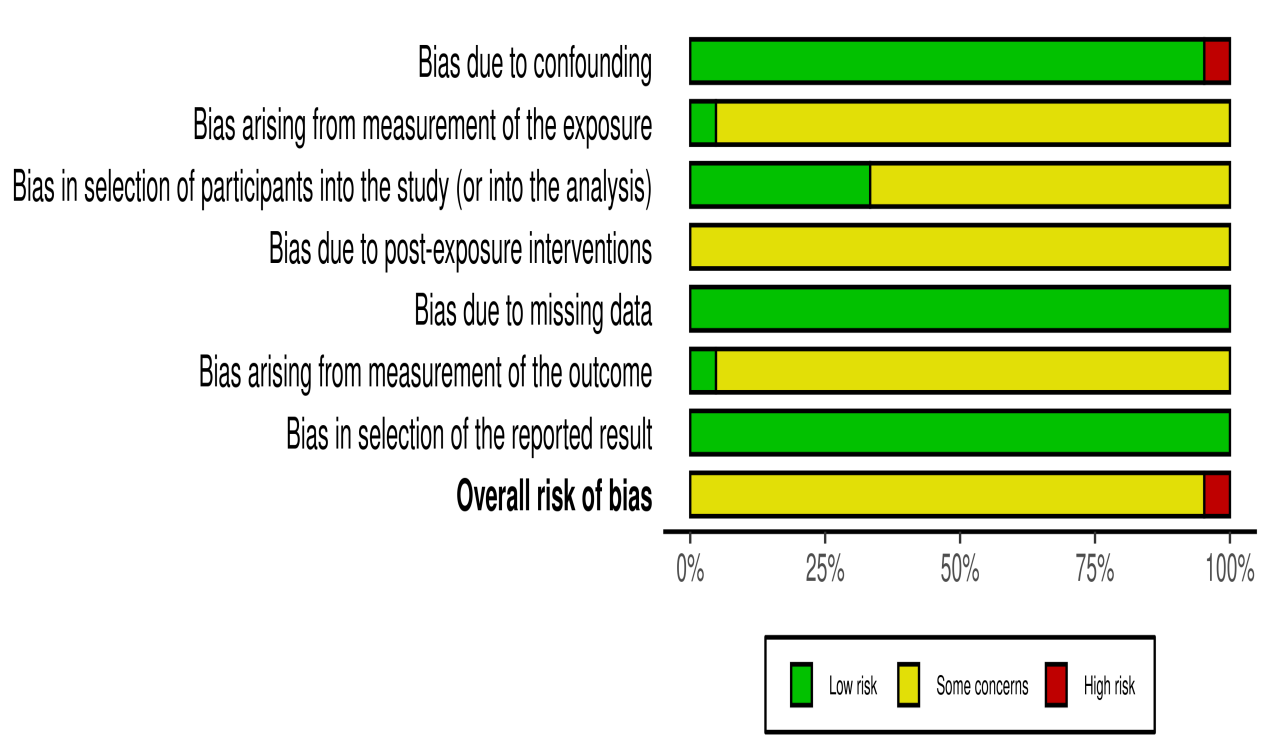


**Supplementary Figure 9.** Overall summary of risk of bias by domain among included studies

**
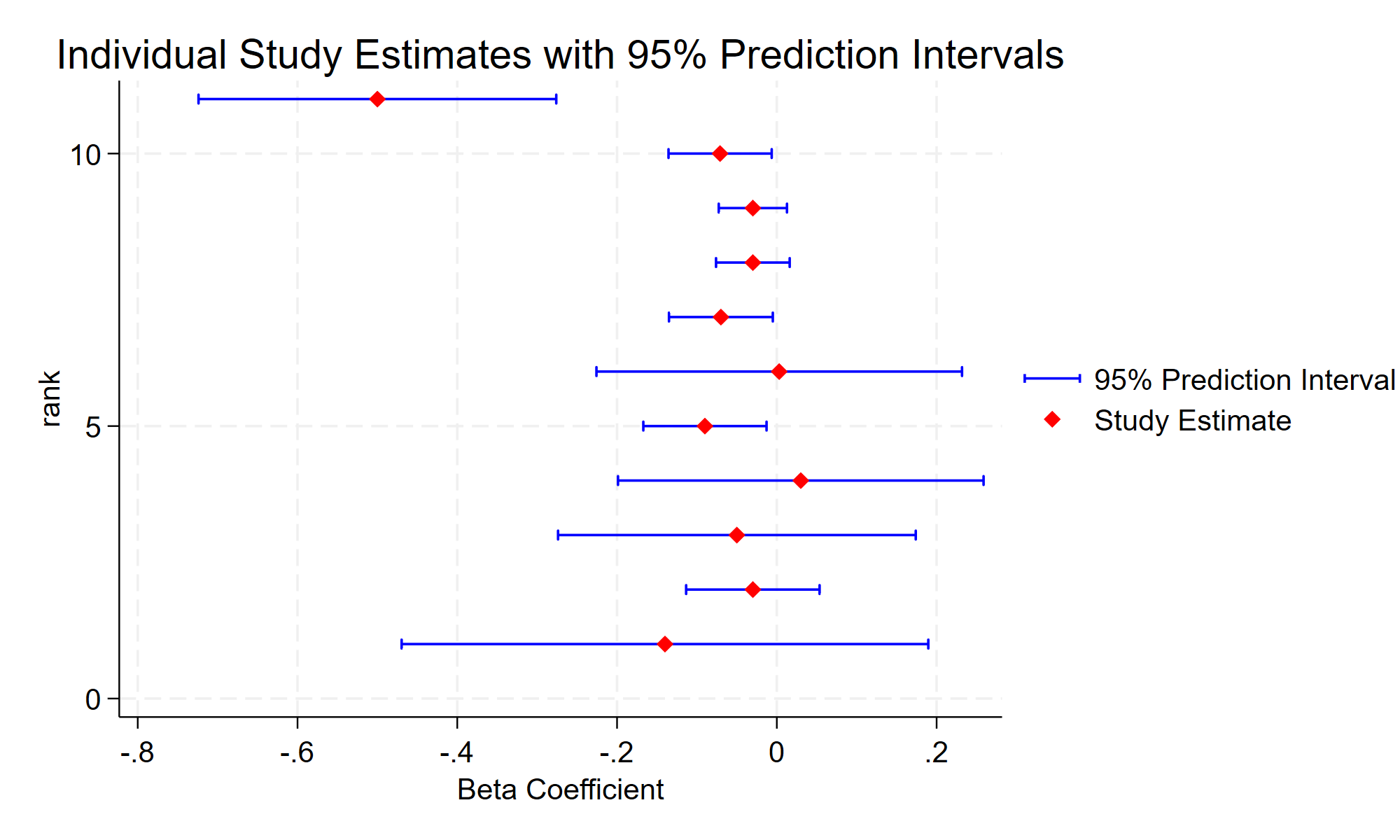
**

**Supplementary Figure 10.** Individual study effect sizes with 95% prediction intervals for the association between screen time and sleep quality (continuous outcomes).


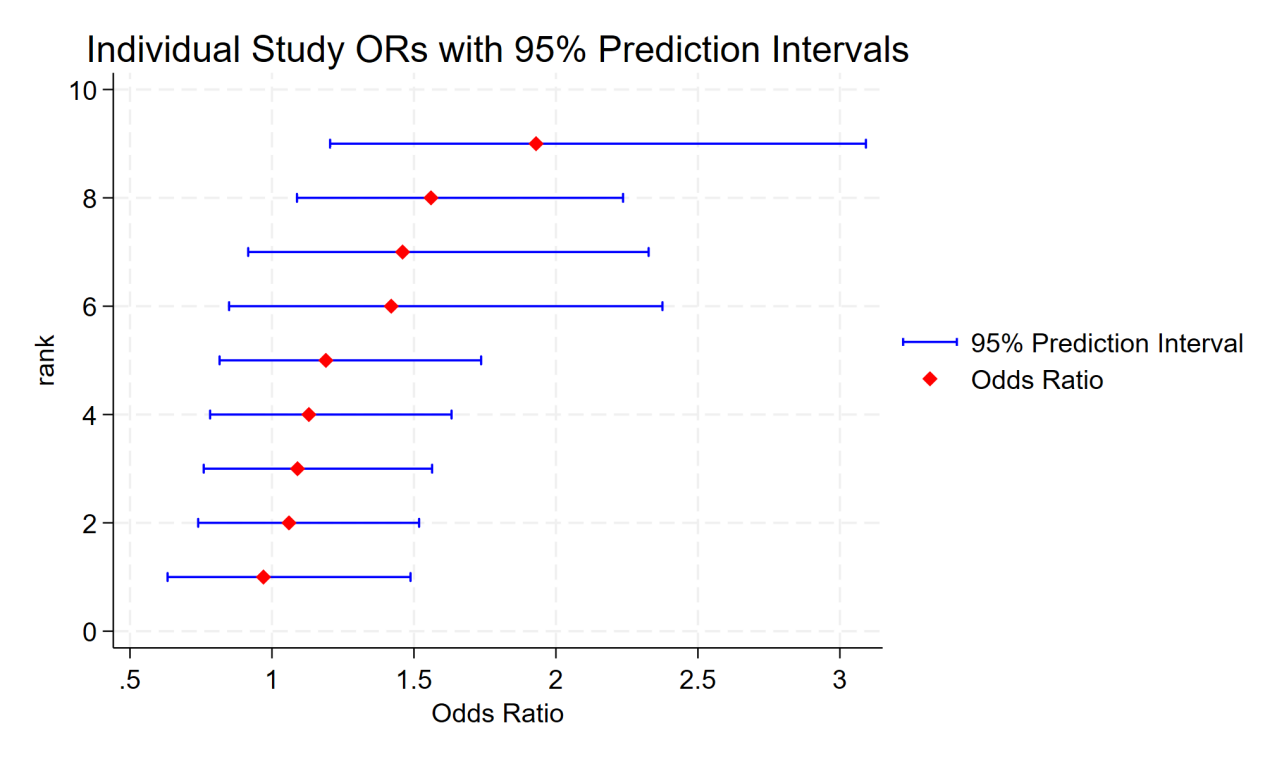


**Supplementary Figure 11.** Individual study effect sizes with 95% prediction intervals for the association between screen time and sleep quality (continuous outcomes).

**
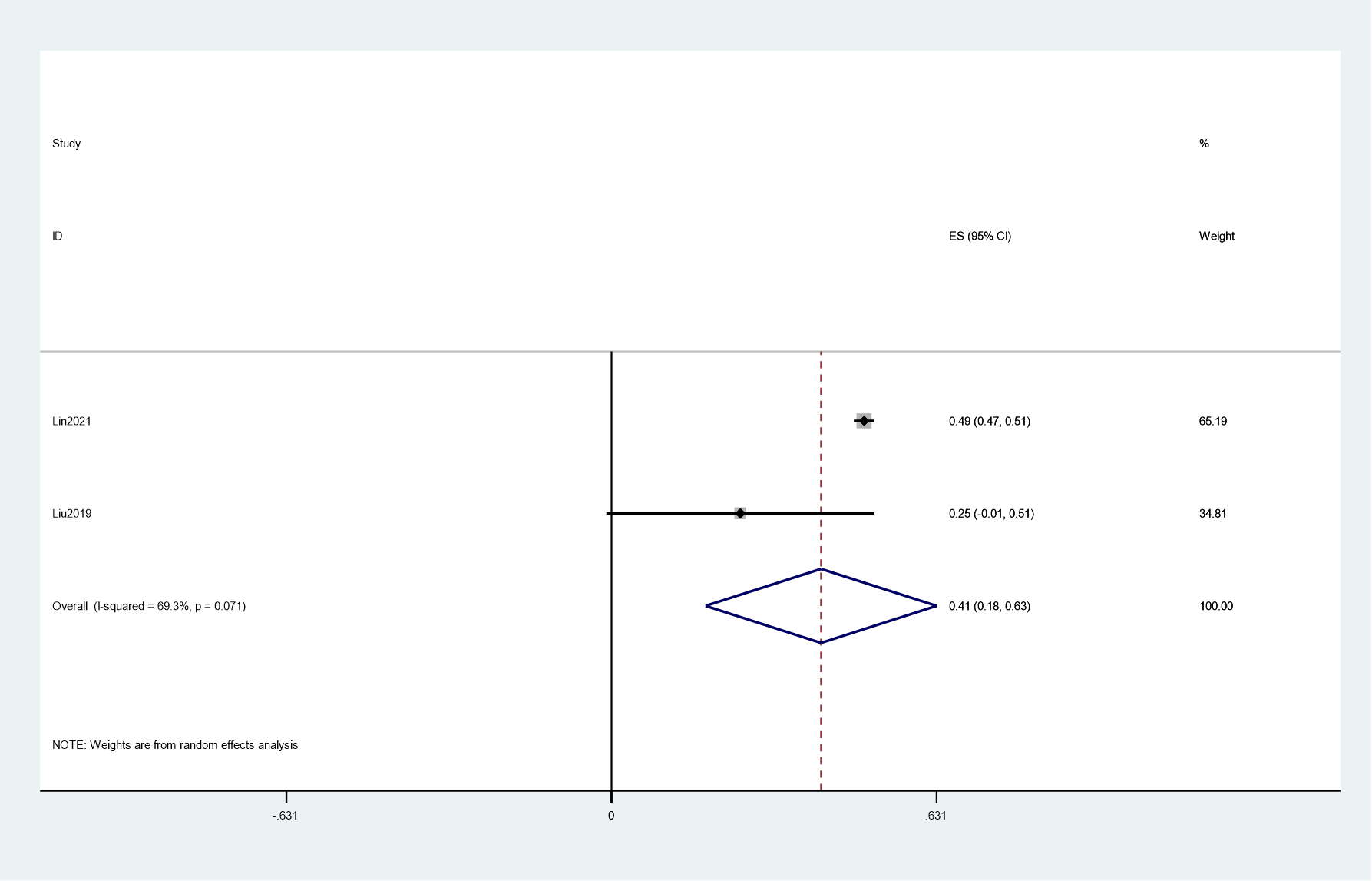
**

**Supplementary Figure 12** Forest plot of insomnia for screen time(Random effects model)


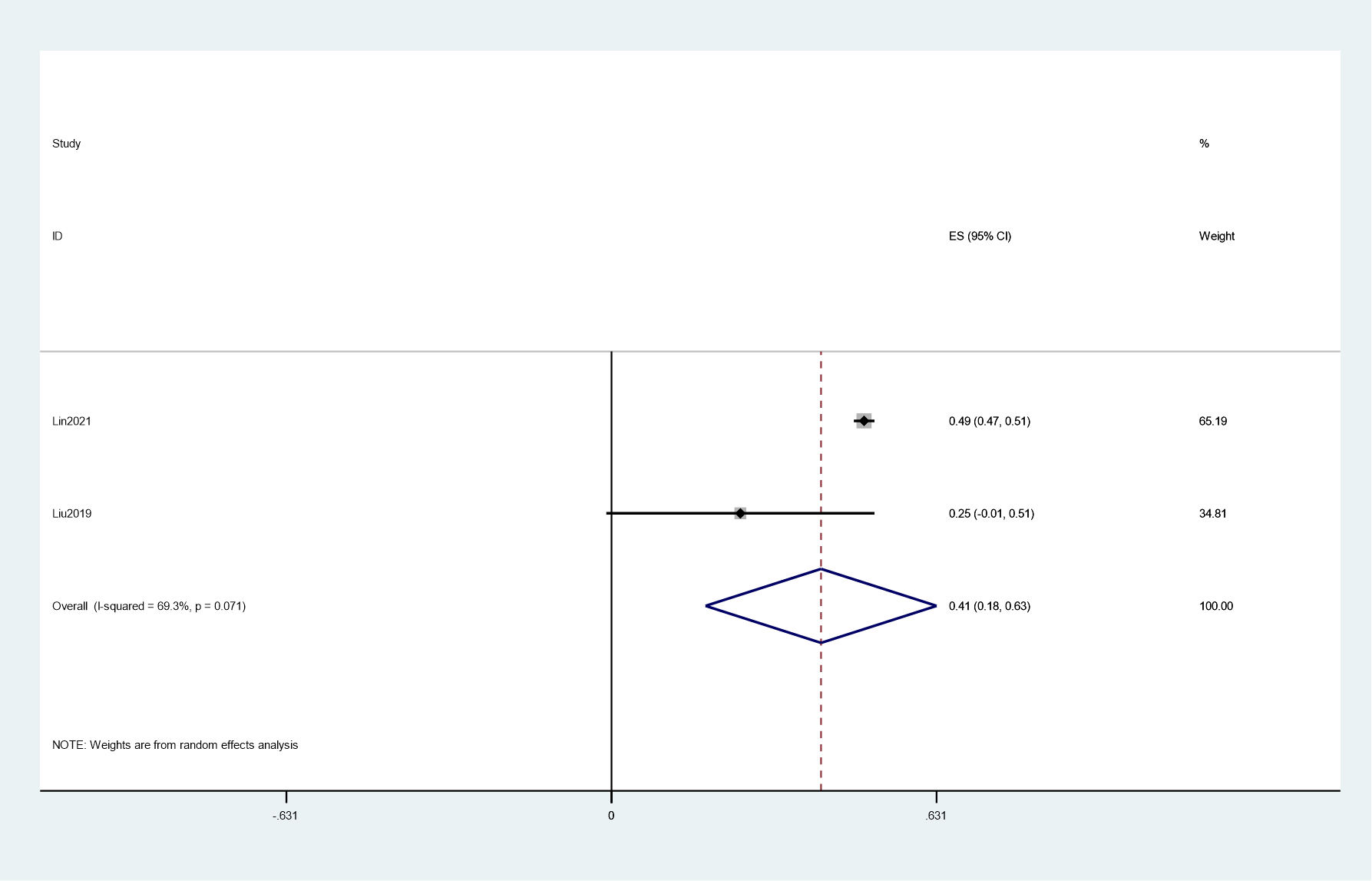


**Supplementary Figure 13** Forest plot of insomnia for screen time(Fixed effects model)
