# Supplementary material for: The association of screen time and the risk of sleep outcomes: a systematic review and meta-analysis
Source: Front Psychiatry. 2025 Dec 17;16:1640263. doi: 10.3389/fpsyt.2025.1640263 (PMC12754674; doi:10.3389/fpsyt.2025.1640263)
Supplement: Supplementary file 1 [file DataSheet1.zip › Supplementary Material 1.docx]

**Supplementary Material 1. – Search Strategy**

**Database**: PubMed

**Date**: 2025.05.06

**Search Strategy:**

| # | Searches |
| --- | --- |
| #1 | (sleep[MeSH Terms]) OR (sleep wake disorders[MeSH Terms]) OR (Sleep Deprivation[MeSH Terms]) |
| #2 | (sleep[Title/Abstract]) OR (sleep wake disorders[Title/Abstract]) OR (sleep deprivation[Title/Abstract]) OR (sleep quality[Title/Abstract]) OR (sleep duration[Title/Abstract]) OR (sleep latency[Title/Abstract]) OR (sleep onset latency[Title/Abstract]) |
| #3 | (dyssomnias[MeSH Terms]) OR (wakefulness[MeSH Terms]) OR (sleep wake disorders[MeSH Terms]) OR (sleep initiation and maintenance disorders[MeSH Terms]) OR (sleep deprivation[MeSH Terms]) OR (insomn*[Title/Abstract]) OR (parasomn*[Title/Abstract]) |
| #5 | screen time[MeSH Terms] |
| #6 | (screen time[Title/Abstract]) OR (screentime[Title/Abstract]) OR (tv time[Title/Abstract]) OR (television time[Title/Abstract]) OR (computer time[Title/Abstract]) OR (video time[Title/Abstract]) OR (screening time[Title/Abstract]) |
| #7 | ("ipad*"[Title/Abstract] OR "cell phone*"[Title/Abstract] OR "mobile phone*"[Title/Abstract] OR "phone*"[Title/Abstract] OR "iphone*"[Title/Abstract] OR "television*"[Title/Abstract] OR "TV"[Title/Abstract] OR "computer*"[Title/Abstract]) |
| #8 | ("electronic game*"[Title/Abstract] OR "computer game*"[Title/Abstract] OR "video game*"[Title/Abstract] OR "social media"[Title/Abstract] OR "screen media"[Title/Abstract] OR "internet use"[Title/Abstract] OR "video viewing"[Title/Abstract]) |
| #9 | （"sedentary behaviour"[Title/Abstract] OR "sedentary time"[Title/Abstract] OR "sedentary times"[Title/Abstract] OR "sedentary activity"[Title/Abstract] OR "sitting time"[Title/Abstract] OR "screen exposure"[Title/Abstract]) |
| #11 | ("cohort studies"[MeSH Terms] OR "longitudinal studies"[MeSH Terms] OR "predict*"[Title/Abstract] OR "associat*"[Title/Abstract] OR "risk"[Title/Abstract] OR "risk"[MeSH Terms] OR "regression*"[Title/Abstract] OR "survival analysis"[Title/Abstract] OR "survival analysis"[MeSH Terms] OR "regression analysis"[MeSH Terms]) |

_­­_

**Database**: EMBASE

**Date**: 2025.05.06

**Search Strategy:**

| # | Searches |
| --- | --- |
| #1 | 'sleep'/exp OR 'sleep disorder'/exp OR 'sleep deprivation'/exp |
| #2 | 'sleep':ab,ti OR 'sleep disorder':ab,ti OR 'sleep deprivation':ab,ti OR 'sleep quality':ab,ti OR 'sleep duration':ab,ti OR 'sleep time':ab,ti OR 'sleep latency':ab,ti OR 'sleep onset latency':ab,ti |
| #3 | 'dyssomnia'/exp OR 'insomnia'/exp OR 'wakefulness'/exp |
| #4 | 'sleep initiation/maintenance disorder':ab,ti OR 'sleep wake disorders':ab,ti OR 'wakefulness':ab,ti OR insomn*:ab,ti OR parasomn*:ab,ti |
| #6 | 'screen time'/exp |
| #7 | 'screentime':ab,ti OR 'screen time':ab,ti OR 'tv time':ab,ti OR 'television time':ab,ti OR 'computer time':ab,ti OR 'video time':ab,ti OR 'screening time':ab,ti |
| #8 | 'eletronic game':ab,ti OR 'computer game*':ab,ti OR 'video ganme*':ab,ti OR 'social media':ab,ti OR 'screen media':ab,ti OR 'internet use':ab,ti OR 'video viewing':ab,ti |
| #9 | 'sedentary behaviour':ab,ti OR 'sedentary time':ab,ti OR 'sedentary times':ab,ti OR 'sedentary activity':ab,ti OR 'sitting time':ab,ti OR 'screen exposure':ab,ti |
| #10 | #5 OR #6 OR #7 OR #8 OR #9 |
| #11 | 'cohort analysis'/exp OR 'risk'/exp OR 'survival analysis'/exp OR 'longitudinal study'/exp OR 'regression analysis'/exp OR 'longitudinal studies':ab,ti OR 'cohort analysis':ab,ti OR 'cohort studies':ab,ti OR risk:ab,ti OR 'survival analysis':ab,ti OR 'regression analysis':ab,ti OR predict*:ab,ti OR associat*:ab,ti OR association:ab,ti OR longitudinal:ab,ti OR cohort:ab,ti OR cohorts:ab,ti OR 'follow up':ab,ti OR 'nested case control study':ab,ti OR 'case cohort':ab,ti |

**Database:** Web of science

**Date**: 2025.05.06

**Search Strategy:**

| # | Searches | Results |
| --- | --- | --- |
| #1 | TS=("sleep" OR "sleep wake disorders" OR "sleep quality"OR"sleep duration"OR"sleep latency"OR"sleep onset latency") | 338,336 |
| #2 | TS=("wakefulness" OR "sleep initiation and maintenance disorders" OR "sleep wake disorders"OR"sleep deprivation"OR"sleep latency"OR"dyssomnias") | 341,312 |
| #3 | #1 OR #2 | 341,312 |
| #4 | TS=("screen time" OR "screentime" OR "tv time"OR"television time"OR"computer time"OR"video time"OR"screening time") | 11,199 |
| #5 | TS=("ipad" OR "cell phone" OR "mobile phone"OR"phone"OR"iphone"OR"television"OR"TV"OR"computer") | 1,216,087 |
| #6 | TS=("social media " OR "screen media" OR "internet use"OR"electronic game"OR"computer game"OR"video game"OR"video viewing") | 163,470 |
| #7 | TS=(" sedentary behaviour" OR " sedentary time" OR " sedentary times "OR"sedentary activity "OR" sitting time"OR"screen exposure") | 12,641 |
| #8 | #4 OR #5 OR #6 OR #7 | 1,378,817 |
| #9 | TS=(”Cohort Studies” OR ”Longitudinal Studies” OR ”longitudinal”OR”cohort”OR”cohorts”OR”follow-up”OR”case-cohort” OR ”predicy*”OR”associat*”OR”risk” OR ”regression *”OR”survival analysis”OR”Regression Analysis”) | 13,324,766 |

**Database**: PsycInfo

**Date**: 2025.05.06

**Search Strategy:**

| # | Searches |
| --- | --- |
| #1 | (sleep or Sleep Wake Disorders or sleep deprivation).mf. |
| #2 | (sleep or Sleep Wake Disorders or sleep deprivation or sleep quality or sleep duration or sleep latency or sleep onset latency).ab. or (sleep or Sleep Wake Disorders or sleep deprivation or sleep quality or sleep duration or sleep latency or sleep onset latency).ti. |
| #3 | (insomnia or wakefulness).mf. |
| #4 | (insomn* or dyssomni* or wakefulness or parasomn* or (Sleep Initiation and Maintenance Disorders)).ab. or insomn*.ti. or dyssomni*.ti. or wakefulness.ti. or parasomn*.ti. or (Sleep Initiation and Maintenance Disorders).ti. |
| #6 | screen time.mf. |
| #7 | (screen time or screentime or television time or computer time or video time or screening time or ipad* or cell phone* or mobile phone* or phone* or iphone* or television* or TV or computer* or electronic game* or computer game* or video game* or social media or screen media or sedentary*).ab. or (screen time or screentime or television time or computer time or video time or screening time or ipad* or cell phone* or mobile phone* or phone* or iphone* or television* or TV or computer* or electronic game* or computer game* or video game* or social media or screen media or sedentary*).ti. |
| #9 | (cohort studies and longitudinal studies) or risk or survival analysis or regression analysis).mf. or (predict* and associat*) or risk or regression* or survival analysis).ab. or (predict* and associat*) or risk or regression* or survival analysis).ti. |
