# Supplementary material for: The association of screen time and the risk of sleep outcomes: a systematic review and meta-analysis
Source: Front Psychiatry. 2025 Dec 17;16:1640263. doi: 10.3389/fpsyt.2025.1640263 (PMC12754674; doi:10.3389/fpsyt.2025.1640263)
Supplement: Supplementary file 1 [file DataSheet1.zip › Supplementary Material 2.docx]

Supplementary Materia 2 . Definition of Sleep Outcomes

1.**Sleep duration:** Total amount of sleep obtained within a 24-hour period, representing the quantitative aspect of an individual’s sleep health^[1]^.

**2.Insomnia:** Difficulty initiating or maintaining sleep, or nonrestorative sleep, despite adequate opportunity, accompanied by daytime impairment^[2]^.

**3.Difficulty initiating sleep:** Trouble falling asleep after going to bed, typically taking more than 30 minutes on most nights^[3]^.

**4.Difficulty maintaining sleep:** Repeated awakenings after sleep onset with trouble returning to sleep, resulting in fragmented or nonrestorative sleep^[3]^.

**5.Sleep difficulties:** Problems related to initiating sleep, maintaining sleep, or experiencing nonrestorative sleep, which may cause daytime impairment such as fatigue, mood disturbances, or reduced cognitive performance^[1]^.

**6.Social jetlag:** Mismatch between an individual’s biological circadian rhythm and social obligations, typically measured as the difference in midpoint of sleep between workdays and free days^[4]^.

**7.Daytime sleep:** Sleep episodes that occur during an individual’s habitual waking period, outside the main nighttime sleep. Daytime napping was defined as daytime sleep episodes between 9 am and 7 pm^[5]^.

**8.Nighttime awakenings:** Episodes of waking up during the main nighttime sleep period, whether brief or prolonged^[6]^.

**9.Bedtime:** Time at which an individual goes to bed with the intention to sleep, marking the beginning of the main sleep period^[7]^.

**10.Short sleep:** Short sleep was defined according to established recommendations from the National Sleep Foundation (NSF)^[8]^ and the American Academy of Sleep Medicine (AASM)^[9-10]^, which provide age-specific thresholds for optimal sleep duration. These thresholds were used to determine whether studies used guideline-aligned definitions in subgroup analyses.National Sleep Foundation (NSF) Recommendations:

(1)Infants (4–11 months): <12 hours;(2)Toddlers (1–2 years): <11 hours;(3)Preschoolers (3–5 years): <10 hours;(4)School-aged children (6–13 years): <9 hours;(5)Teenagers (14–17 years): <8 hours;(6)Young adults and adults (18–64 years): <7 hours;(7)Older adults (65+ years): <7 hours.American Academy of Sleep Medicine (AASM) Guidelines:(1)Children (6–12 years): <9 hours(2)Teenagers (13–18 years): <8 hours(3)Adults (≥18 years): <7 hours.

**Reference**

[1] Buysse DJ. Sleep health: can we define it? Does it matter? Sleep. 2014;37(1):9–17. doi:10.5665/sleep.3298

[2] Seow LSE, Verma SK, Mok YM, Kumar S, Chang S, Satghare P, Hombali A, Vaingankar J, Chong SA, Subramaniam M. Evaluating DSM-5 Insomnia Disorder and the Treatment of Sleep Problems in a Psychiatric Population. J Clin Sleep Med. 2018;14(2):237–244. doi:10.5664/jcsm.6942

[3] American Academy of Sleep Medicine. International classification of sleep disorders: Third edition, text revision (ICSD-3-TR). Darien, IL: Author; 2023. Available from: https://aasm.org/clinical-resources/international-classification-of-sleep-disorders-3rd-edition-icsd-3/

[4] Wittmann M, Dinich J, Merrow M, Roenneberg T. Social jetlag: Misalignment of biological and social time. Chronobiol Int. 2006;23(1-2):497–509. doi:10.1080/07420520500545979

[5] Gao C, Zheng X, Cai R, Yu L, Schneider JA, Buchman AS, Bennett DA, Leng Y, Ibáñez A, Gao L, Hu K, Li P. Timing and intraindividual variability of daytime napping and Alzheimer's disease in older adults. Commun Med. 2025;5(1):219. doi:10.1038/s43856-025-00936-1

[6] Ohayon MM, Krystal A, Roehrs TA, Roth T, Vitiello MV. Using difficulty resuming sleep to define nocturnal awakenings. Sleep Med. 2010;11(3):236–241. doi:10.1016/j.sleep.2009.11.004

1. Olds TS, Maher CA, Matricciani L. Sleep duration or bedtime? Exploring the relationship between sleep habits and weight status and activity patterns. Sleep. 2011;34(10):1299–1307. doi:10.5665/SLEEP.1266
2. Hirshkowitz M, Whiton K, Albert SM, Alessi C, Bruni O, DonCarlos L, et al. National Sleep Foundation’s sleep time duration recommendations: methodology and results summary. Sleep Health. 2015;1(1):40–43. doi:10.1016/j.sleh.2014.12.010
3. Watson NF, Martin JL, Wise MS, Carden KA, Curhan D, Consensus Conference Panel. Joint consensus statement of the American Academy of Sleep Medicine and Sleep Research Society on the recommended amount of sleep for a healthy adult: methodology and discussion. J Clin Sleep Med. 2015;11(8):931–952. doi:10.5664/jcsm.4950
4. Paruthi S, Brooks LJ, D’Ambrosio C, Hall WA, Kotagal S, Lloyd RM, et al. Consensus statement of the American Academy of Sleep Medicine on the recommended amount of sleep for healthy children: methodology and discussion. J Clin Sleep Med. 2016;12(11):1549–1561. doi:10.5664/jcsm.6288
