# Supplementary material for: The association of screen time and the risk of sleep outcomes: a systematic review and meta-analysis
Source: Front Psychiatry. 2025 Dec 17;16:1640263. doi: 10.3389/fpsyt.2025.1640263 (PMC12754674; doi:10.3389/fpsyt.2025.1640263)
Supplement: Supplementary file 1 [file DataSheet1.zip › Supplementary Table 1.docx]

**Supplementary Table 1 .** List of articles that were excluded after full-text assessment

| **Title** | **Author Year** | **Reasons for exclusion** |
| --- | --- | --- |
| Use of social media is associated with short sleep duration in a dose–response manner in students aged 11 to 20 years | Sampasa-Kanyinga 2018 | Study design:cross-sectional |
| Trends in sleeping difficulties among European adolescents: Are these associated with physical inactivity and excessive screen time? | Ghekiere 2018 | Study design:cross-sectional |
| The role of sleep in prospective associations between parent reported youth screen media activity and behavioral health | Rojo-Wissar 2023 | Study design:cross-sectional |
| The longitudinal interaction of adolescents’ interest in physical education, school burnout, and disturbed sleep related to social media and phone use | Akungu 2021 | Exposure(did not meet inclusion criteria) |
| The impact of screen time changes on anxiety during the COVID‑19 pandemic: Sleep and physical activity as mediators | Chen 2022 | Exposure:without a clearly defined amount of screen time |
| The associations of parental COVID‐19 related worries, lifestyles, and insomnia with child insomnia during the COVID‐19 outbreak | Zhan 2022 | Study design:Cross-sectional |
| Sleep deprived but socially connected: balancing the risks and benefits of adolescent screen time during COVID-19 | Fry 2021 | Study design:Review |
| Sedentary behaviors, psychological status, and sleep quality in Chinese university students | Wang 2019 | Study design:Cross-sectional |
| Screen time use impacts low‐income preschool children's sleep quality, tiredness, and ability to fall asleep. | Waller 2021 | Study design:Cross-sectional |
| Screen time and toddlers: New evidence on potentially negative effects | Beal 2020 | Study design:Review |
| Relationship between screentime and depressive symptoms and associated mediators for African American adolescents in the United States | Jones 2023 | Study design:Cross-sectional |
| Reducing the use of screen electronic devices in the evening  is associated with improved sleep and daytime vigilance in  adolescents | Perrault 2019 | Study design is not a cohort study. |
| Physical activity, screen-based sedentary behavior, and sleep duration in adolescents: Youth risk behavior survey, 2011–2013 | Kim 2016 | Study design:Cross-sectional |
| Physical activity, screen time, and sleep trajectories from childhood to adolescence: The influence of sex and body weight status | Guimaraes 2021 | Data not meeting inclusion criteria |
| Parent–adolescent patterns of physical activity, sedentary behaviors and sleep among a sample of overweight and obese adolescents | Tu 2015 | Study design:Cross-sectional |
| Impact of pre-sleep habits on adolescent sleep: An Italian population-based study | Nosetti 2021 | Study design:Cross-sectional |
| Associations among toddlers’ and preschoolers’ sleep problems, emotional reactivity, sleep regime and parental applied rules for screen-based media use | Baukiene 2020 | Study design:Cross-sectional |
| Gender‐specific factors associated with shorter sleep duration at age 3 years | Plancoulaine 2015 | Study design:Cross-sectional |
| Association between Televison Viewing and Insomnia During Early Adolescence | Cronlein 2007 | Study design:Review |
| Changes in lifestyle behaviours during the COVID‐19 confinement in Spanish children: A longitudinal analysis from the MUGI project | Medrano 2020 | Outcome(did not meet inclusion criteria) |
| Association between electronic media use and sleep habits: An eight-day follow-up study | Kubiszewski 2013 | Study design:Cross-sectional |
| Physical Activity, Screen-Based Sedentary Behavior, and Sleep Duration in Adolescents: Youth Risk Behavior Survey, 2011-2013 | Kim 2016 | Study design:Cross-sectional |
| Sleep, Anxiety, and Academic Performance: A Study of Adolescents From Public High Schools in China | Zhang 2021 | Study design:Cross-sectional |
| Meeting new Canadian 24-Hour Movement Guidelines for the Early Years and associations with adiposity among toddlers living in Edmonton, Canada | Lee 2017 | Study design:Cross-sectional |
| An Exploration of Domain-Specific Sedentary Behaviors in College Students by Lifestyle Factors and Sociodemographics | Carpenter 2021 | Study design:Cross-sectional |
| Clusters of diet, physical activity, television exposure and sleep habits and their association with adiposity in preschool children: the EDEN mother-child cohort | Saldanha-Gomes 2020 | Outcome (did not meet inclusion criteria) |
| School start time changes in the COMPASS study: associations with youth sleep duration, physical activity, and screen time | Karen 2018 | Outcome (did not meet inclusion criteria) |
| Physical Activity, Screen Time, and Sleep Trajectories From Childhood to Adolescence: The Influence of Sex and Body Weight Status | Guimaraes 2020 | Outcome(did not meet inclusion criteria) |
| Study on lifestyle habits affecting sleep disorders at the undergraduate education stage in Xuzhou City, China | Wu 2022 | Study design:Cross-sectional |
| Health Behavior Changes during the COVID-19 Pandemic: A Longitudinal Analysis among Children | Bekelman 2022 | Outcome/exposure (did not meet inclusion criteria) |
| Screen use, sleep duration, daytime somnolence, and academic failure in school-aged adolescents | Perez-chada 2023 | Study design:Cross-sectional |
| Daily touchscreen use in infants and toddlers is associated with reduced sleep and delayed sleep onset | Cheung 2017 | Study design:Cross-sectional |
| Processed data on the night-time use of screen-based media devices and adolescents' sleep quality and health-related quality of life | Mireku 2019 | Study design:Cross-sectional |
| Late-Night Digital Media Use in Relation to Chronotype, Sleep and Tiredness on School Days in Adolescence | Kortesoja 2023 | Study design:Cross-sectional |
| Longitudinal associations between time spent using technology and sleep duration among adolescents | Mazzer 2018 | Outcome/exposure (did not meet inclusion criteria) |
| Long-term effect of mobile phone use on sleep quality: Results from the cohort study of mobile phone use and health (COSMOS) | Tettamanti 2020 | Exposure:RF-EMF exposure |
| Relating Use of Mobile Phones to Reported Sleep Quality | Herr 2005 | Study design:Cross-sectional |
| Sleep patterns, eating habits and electronic media exposure among adolescents | Shochat 2009 | Study design:Cross-sectional |
| Impact of television-watching and computer-using on sleep patterns and sleep problems of school-aged children in Shanghai | Li 2009 | Study design:Cross-sectional |
| Brief report: Predictors of heavy Internet use and associations with health-promoting and health risk behaviors among Hong Kong university students | Kim 2010 | Study design:Review |
| Sleep disturbance in german adolescents: Associated risks and resources | Cohrs2010 | Study design:Review |
| Associations between media use in the bedroom, sleep habits and symptoms of insomnia | Brunborg 2010 | Study design:Review |
| Teen sleep, media exposures, and physical activity: Results from the 2007 and 2009 youth risk behavior surveys | Fitzgerald 2011 | Study design:Review |
| Sedentary behaviours and inflammatory status in children and adolescents | Gonzalez 2011 | Study design:Review |
| Media usage as a predictor of irregular sleeping patterns | Teija 2011 | Study design:Review |
| sleep behavior and risk factors for deterioration of sleep patterns among Japanese middle-school students | Shigeta 2011 | Study design:Review |
| Physical activity, sedentary behaviour and sleep in Canadian children: parent-report versus direct measures and relative associations with health risk | Colley 2012 | Study design:Cross-sectional |
| Adolescents' media use and its effect on sleep | Davis 2012 | Study design:Review |
| Teen sleep, media exposure, and physical activity: Results from the 2009 youth risk behavior survey | Fitzgerald 2011 | Study design:Cross-sectional |
| Impact of reading or playing a video-game before going to bed on adolescent sleep | Blau 2012 | Study design:Review |
| Do reading or playing a video-game before going to bed influence sleep? | Fietze 2012 | Study design:Review |
| Impact of television on sleep habits | Nag 2012 | Study design:Cross-sectional |
| High incidence of excessive daytime sleepiness in high-school students | Jain 2012 | Study design:Cross-sectional |
| What is the association between screen time and outcomes for canadian children? | Tough 2012 | Study design:Review |
| Technology use of australian high school students and its association with sleep duration and sleep and wake times: The australian broadcasting corporation's national science week big sleep survey | Gamble 2012 | Study design:Review |
| Multiple behavioral factors related to weight status in a sample of early adolescents: Relationships of sleep, screen time, and physical activity | Steele 2012 | Study design:Cross-sectional |
| Sleep-wake habits: Associations with weight status, physical activity and screen-time in British 11-16 year olds | Sandercock 2012 | Study design:Review |
| Relations of childhood stress with diet,sleep,screen time and obesity: The chibs survey | Michels 2013 | Study design:Review |
| Behavioral predictors of screen time among high school students in malden, ma | Dube 2013 | Study design:Review |
| Sleep and physical activity pattern of obese children | julia2013 | Study design:Cross-sectional |
| Impact of the pediatric residents' initiative for healthy active living in youth (RHALY): A prospective cohort study | Khalifah 2014 | Study design:Review |
| Availability and use of electronic media is associated with shorter sleep duration and poorer sleep quality in school-aged children | Paquin 2014 | Study design:Review |
| The relationship between television viewing and sleep duration | Basner 2014 | Data not meeting inclusion criteria |
| The impact of demographics and sleep hygiene factors on sleep in children aged 2-10 years | Richdale 2014 | Study design:Review |
| Snoring, sleep and TV time in a school-aged cohort of inner-city asthmatics | Wright 2014 | Exposure (did not meet inclusion criteria) |
| Television, sleep, outdoor play and BMI in young children: the GECKO Drenthe cohort | Sijtsma 2014 | Data not meeting inclusion criteria |
| Daily monitoring of mood, sleep behaviours, screen time, energy levels and physical activity in a sample of depressed youth | Giles 2014 | Study design:Cross-sectional |
| Past-day recall of sedentary time: Validity of a self-reported measure of sedentary time in a university population | Clark 2016 | Outcome (did not meet inclusion criteria) |
| How to get a better night's sleep: Be active and reduce sedentary behaviour | Kakinami 2015 | Study design:Cross-sectional |
| Determinants of young children's sleep duration in the U.S. affiliated pacific: The children's healthy living program | Li 2015 | Study design:Cross-sectional |
| Lifestyle and working memory in children | Lopez-Vicente 2015 | Study design:Review |
| What's keeping teenagers awake? relationships and mechanisms between pre-bedtime behaviors and sleep in adolescents during school and vacation | Harbard 2016 | Study design:Cross-sectional |
| Association between habitual activity and knowledge of public  health guidelines in Canada | Leblanc 2015 | Study design:Review |
| Does access to and use of TVs, computers, tablets, video games and cellphones before bedtime affect sleep and body weight of children? | Veugelers 2016 | Study design:Review |
| Television and computer hours and sleep disturbances: In the 2005-2006 National Health and Nutrition Examination Survey | Lewis 2016 | Study design:Review |
| The adverse effects of mobile device-based electronic and social media use on sleep and mood in a group of American university students | Polos 2016 | Study design:Review |
| Sleep patterns, sleep habits and screen exposure: Cultural differences among Jewish and Muslim school-aged children in Israel | Haimov 2016 | Study design:Review |
| Do lifestyle habits influence the development of the metabolically healthy obese phenotype in youth | Henderson2016 | Study design:Review |
| Changes in sleep duration and recreational screen time among Canadians, 1998–2010 | Leech 2016 | Study design:Cross-sectional |
| The impact of electronic media and school schedule on sleep of adolescents | Konsta 2017 | Study design:Review |
| Bedtime use of technology: Effects on sleep in children and adolescents | Fuller 2018 | Study design:Review |
| Influences of screen media use near bedtime on daytime sleepniess and self-satisfaction among college students: The mediating effect of valid sleep time | Yang 2018 | Study design:Review |
| Sleep and exposure to screens of digital media devices in Israel | Green 2019 | Study design:Review |
| Sedentary behavior and sleep in toddlers: Within and between subject effects | Armstrong 2018 | Study design:Review |
| Insomnia symptoms and sleep duration mediate the association between adolescent screen time and depressive symptoms | Li 2018 | Study design:Review |
| Exposure to screens of digital media devices, sleep, and concentration abilities in a sample of Israel adults | Green 2018 | Study design:Cross-sectional |
| Screen time exposure and sleep among children with developmental disabilities | Aishworiya 2018 | Study design:Cross-sectional |
| Digital media use before bedtime and sleep quality among Finnish adolescents | Paarnio 2023 | Study design:Review |
| Sleep mediates the association between school pressure, physical activity, screen-time and psychological distress in adolescents | Dhondt 2018 | Study design:Review |
| Screen time exposure and sleep among children with ADHD | Matias 2019 | Study design:Review |
| Early childhood digital media use: Inverse associations with sleep time consistency and sleep duration | Rapoport 2019 | Study design:Review |
| Association of depression and excessive daytime sleepiness among sleep-deprived college freshmen in northern Taiwan | Tsou 2019 | Study design:Cross-sectional |
| Social media use is associated with sleep duration and disturbance among adolescents in Bangladesh | Khan 2019 | Study design:Cross-sectional |
| Media Use and Sleep in Teenagers: What Do We Know? | Hale 2019 | Study design:Review |
| SMARTPHONE USE AND SLEEP IN CHILDREN | Kim 2019 | Study design:Review |
| Sleep and media use in 3-6 year-old children: differences between good and poor sleepers | Wong 2019 | Study design:Review |
| To investigate the impact of sleep deprivation related to the use of screen-based media before sleep in neurocognitive function among healthy teenagers: a preliminary study | Jan 2019 | Study design:Review |
| Excessive internet use and sleep | Ohayon 2019 | Study design:Review |
| Social jet lag, screen time, physical activity and sleep quality in Indian young adults | Aggarwal2019 | Study design:Review |
| Screen time and sleep: let there not be light! | Ranganathan 2019 | Study design:Review |
| Media use and short sleep duration among children aged 3-17 years-national survey of children's health, 2016-2017 | Wheaton 2020 | Study design:Review |
| Electronic media use is associated with poor sleep in 3-6 year-old children | Wong 2020 | Study design:Review |
| Social media use and actigraphic measures of sleep timing among high-risk adolescents | Hamilton 2020 | Study design:Review |
| One size does not fit all: Identifying clusters of physical activity, screen time, and sleep behaviour co-development from childhood to adolescence | Gallant 2020 | Data not meeting inclusion criteria |
| The school schedule effect on self-reported sleep length of children and youth in Spain | Gabaldon 2022 | Study design:Review |
| DIET, SLEEP, SCREEN TIME, AND EXERCISE: ASSOCIATIONS WITH CHILD MENTAL HEALTH AT AGE 5 | Tombeau 2020 | Study design:Review |
| Sleep patterns of urban south indian adolescents | Anjali 2020 | Study design:Review |
| Short and long sleep duration and associated factors in pre-adolescence and early adulthood: evidence from the 1993 Pelotas birth cohort study | Wehrmeister 2020 | Study design:Cross-sectional |
| The adverse impact of excessive smartphone screen-time on sleep quality among young adults: A prospective cohort | Arshad 2021 | Data not meeting inclusion criteria |
| Sociodemographic predictors of changes in physical activity, screen time, and sleep among toddlers and preschoolers in chile during the covid-19 pandemic | Farias 2021 | Study design:Cross-sectional |
| Screen use before bedtime: Consequences for nighttime sleep in young children | Staples 2021 | Study design:Cross-sectional |
| Screen-based activities predict delayed sleep timing within and between adolescents | Reichenberger 2021 | Study design:Review |
| Digital media use and sleep in college students during COVID-19 pandemic | Li 2021 | Study design:Review |
| Screen time, sport and sleep of a population of Algerian adolescents – Influence of gender and body mass index | Sersar 2021 | Study design:Cross-sectional |
| Effects of two different types of electronic media use on sleep duration among middle and high school adolescents in Texas | Navazi 2023 | Study design:Review |
| Prevalence of Sleep Practices, Circadian Types and Their Effect on Sleep Beliefs in General Population: Knowledge and Beliefs About Sleep and Sleep Practices (KNOBS Survey) | Devaraj 2021 | Study design:Cross-sectional |
| Adolescents’ digital screen time as a concern for health and well-being? Device type and context matter | Fairclough 2021 | Study design:Review |
| Partial mediating effect of pre-sleep hyperarousal between bedtime mobile phone use and subjective sleep quality among military university students | He 2021 | Study design:Cross-sectional |
| Determinants of dietary behaviour and physical activity in Australian adolescents | Marjanovic 2021 | Study design:Review |
| Physical and social distancing measures and child health behaviours during COVID-19: A cohort study | Li 2021 | Study design:Review |
| Screen time and delayed development in toddlers-is there a link? | Bassi 2021 | Study design:Review |
| he Ethics of Persuasive Design in Technology Used by Children and Adolescents | Owenz 2021 | Study design:Review |
| SPIS-F score:a score based on screen time, physical activity, sleep hours, energy intake and family history of obesity in children aged 5-12 year and its correlation with bmi and childhood obesity in indian children | Arora 2021 | Study design:Review |
| Sleep Patterns, Problems, and Habits Among Egyptian Adolescents with Epilepsy | Khadra 2021 | Study design:Cross-sectional |
| Sleep, screen time and emotional disorders: implications for undergraduates | Mota 2022 | Study design:Review |
| Relation of sleep habits and physical activities with sleep quality | Diogo 2022 | Study design:Review |
| Screen time and sleep status associated with emotional and behavioral problems in preschool children | Chen 2022 | Study design:Cross-sectional |
| Health Issues Associated With Increased Smart Phone Usage Among Students During Covid-19 Pandemic Wave 2.0 In India | Raja 2022 | Study design:Cross-sectional |
| Nighttime cell phone use and sleep quality in young adults | Suresh 2022 | Study design:Cross-sectional |
| Child and Parent Physical Activity, Sleep, and Screen Time During COVID-19 and Associations With Mental Health: Implications for Future Psycho-Cardiological Disease? | Olive 2022 | Study design:Cross-sectional |
| 134. The Effect of the COVID-19 Pandemic on the Video Gaming Behavior, Depressive Symptoms, Sleep Quality, and Physical Activity of Excessive Video Gamers | Akbulut 2022 | Study design:Review |
| SCREEN TIME AND INSOMNIA SYMPTOMS IN UNIVERSITY STUDENTS | Hodges 2022 | Study design:Review |
| Sleep-health care and education are urgently needed for excessive screen time and sleep problems among school-age children | Okawa 2022 | Study design:Review |
| How do youth really use their digital devices? Insights from the bedtime and electronic devices (BED) study | Wickham 2022 | Study design:Review |
| Effect of COVID-19 pandemic on sleep quality, sleep duration and acute stress in Indian young adults | Akhtar 2022 | Study design:Review |
| Prevalence of unhealthy lifestyle among children and adolescents of Han nationality in China | Chen 2022 | Study design:Cross-sectional |
| Social Media at night and Sleep Quality : the relevance of FOMO, Cognitive Pre-sleep Arousal and Maladaptive cognitive emotion regulation | Almeida 2022 | Study design:Review |
| Exposure to screens and changes in toddlers’ sleep during COVID era | Hasiyan 2022 | Study design:Review |
| Sleep habits and screen use by adolescents during COVID-19 lockdown | Vieira 2022 | Study design:Review |
| Relationship between time spent playing internet gaming apps and behavioral problems, sleep problems, alexithymia, and emotion dysregulations in children: a multicentre study | Ahmed 2022 | Study design:Cross-sectional |
| Relationship of sleep quality with screen-based sedentary time and physical activity in adolescents — the moderating effect of body mass index | Damato 2022 | Study design:Cross-sectional |
| IMPACT OF DIGITAL SCREEN USE IN RELATION TO DRY EYE SYMPTOMS AND QUALITY OF SLEEP: A STUDY IN TERTIARY CARE CENTRE | Batham 2023 | Study design:Cross-sectional |
| Effects of the COVID-19 Pandemic on Screen Time and Sleep in Early Adolescents | Kiss 2023 | Study design:Cross-sectional |
| Problematic mobile phone use among medical students and professionals: Its impact on sleep quality and depressive symptoms | Finkel 2023 | Study design:Review |
| Sleep hygiene and behavioral problems among children with a parental history of alcoholism | Wong 2023 | Study design:Review |
| SLEEP PATTERNS, IMPACT OF POOR SLEEP, AND DETERMINANTS OF SLEEP QUALITY IN ADOLESCENTS IN THE UK AND INDIA | An 2023 | Study design:Review |
| Effect of Confinement Due to COVID-19 Pandemic in Stress, Sleep Quality, Eating Habits, BMI and Use of Online Platforms of Mexican University Students: A Longitudinal Study | Ochoa-Ruiz 2023 | Study design:Review |
| Screen exposure and sleep: How the COVID-19 pandemic influenced children and adolescents – A questionnaire-based study | Moavero 2023 | Study design:Cross-sectional |
| Health behavior clusters, temperament and weight status among Finnish preschoolers | Vepsalainen 2023 | Study design:Review |
| Study on lifestyle habits affecting sleep disorders at the undergraduate education stage in Xuzhou City, China | Wu 2022 | Study design:Cross-sectional |
| Health-Related Behaviors Among School-Aged Children and Adolescents During the Spanish Covid-19 Confinement | Bueno 2020 | Study design:Cross-sectional |
| Prospective associations of lifestyle patterns in early childhood with sociolect-emotional and behavioral development and BMI: An outcome-wide analysis of the EDEN mother-child cohort | Descarpentrie 2023 | Exposure(not meet inclusion criteria) |
| Timing of puberty in boys and girls: Implications for population health | Hoyt 2020 | Data not meeting inclusion criteria |
| Association Between Internet Use, Sleep, Cognition and Physical Activity Levels During COVID-19 Lockdown | Singla 2023 | Study design:Cross-sectional |
| Predictors of Preadolescent Children's Recreational Screen Time Duration During the COVID-19 Pandemic | McArthur 2022 | Study design:Cross-sectional |
| Short Sleep Duration and Screen-Based Activities: A Longitudinal Multilevel Analysis | Alqaderi 2016 | Data not meeting inclusion criteria |
| Physical Functions among Children before and during the COVID-19 Pandemic: A Prospective Longitudinal Observational Study | Ito 2022 | Outcome:not meet inclusion criteria |
| How Infant and Toddlers' Media Use Is Related to Sleeping Habits in Everyday Life in Italy | Bellagamba 2021 | Study design:Cross-sectional |
| Child and family factors associated with child mental health and well-being during COVID-19 | McArthur 2023 | Outcome:not meet inclusion criteria |
| Lifestyle and mental health disruptions during COVID-19 | Giuntella 2021 | Data not meeting inclusion criteria |
| Associations of adolescents' lifestyle habits with their daytime functioning in Japan | Kohyama 2020 | Study design:Cross-sectional |
| Associations of adolescents’ lifestyle habits with their daytime functioning in Japan | Sun 2009 | Data not meeting inclusion criteria |
| Psychological Effects of Screen Time in Health Care Workers During the COVID-19 Pandemic | Le 2021 | Data not meeting inclusion criteria |
| Differential associations between passive and active forms of screen time and adolescent mood and anxiety disorders | Kim 2019 | Study design:Cross-sectional |
| Television in the bedroom and increased body weight: potential explanations for their relationship among European schoolchildren | Cameron 2012 | Study design:Cross-sectional |
| Insufficient Sleep Duration And Its Association With Breakfast Intake, Overweight/Obesity, Socio-Demographics And Selected Lifestyle Behaviors Among Saudi School Children | Hazzaa 2019 | Study design:Cross-sectional |
| Editorial: Did Goldilocks Have It Right? How Do We Define Too Little, Too Much, or Just Right? | Belfort 2020 | Study design:Review |
| Screen-Based Behaviors of Adolescents in Bangladesh | Khan 2016 | Study design:Cross-sectional |
| Influence of screen time on the sleep quality of students | Nestler 2023 | Study design:Cross-sectional |
| Children's Environmental Health in the Digital Era: Understanding Early Screen Exposure as a Preventable Risk Factor for Obesity and Sleep Disorders | Wolf 2018 | Study design:Review |
| Mental Health, Smartphone Use Type, and Screen Time Among Adolescents in South Korea | Woo 2021 | Study design:Cross-sectional |
| The freshman sleep and health (FRoSH) study: Examining sleep and weight gain in incoming college freshmen | Jaiswal 2022 | Data not meeting inclusion criteria |
| Effect of Screen Time on Physical and Mental Health and Eating Habits During COVID-19 Lockdown in Lebanon | Kamaleddine 2021 | Study design:Cross-sectional |
| Movement behaviors and post traumatic stress disorder during the COVID-19 pandemic: A retrospective study of Chinese university students | Feng 2022 | Study design:Cross-sectional |
| Child and Parent Physical Activity, Sleep, and Screen Time During COVID-19 and Associations With Mental Health: Implications for Future Psycho-Cardiological | Olive 2021 | Data not meeting inclusion criteria |
| Electronic Media Use and Sleep Among Preschoolers: Evidence for Time-Shifted and Less Consolidated Sleep | Beyens 2018 | Study design:Cross-sectional |
| Socio-Economic Status, Time Spending, and Sleep Duration in Indian Children and Adolescents | Radhika 2017 | Study design:Cross-sectional |
| Prevalence and Correlates of Meeting Sleep, Screen-Time, and Physical Activity Guidelines Among Adolescents in the United Kingdom | Pearson 2019 | Study design:Review |
| The impact of sleep, physical activity and sedentary behaviour on symptoms of depression and anxiety before and during the COVID-19 pandemic in a sample of South African participants | Lewis 2021 | Study design:Cross-sectional |
| Mutual relations between sleep deprivation, sleep sealers and risk behaviour in adolescents | Paiva 2016 | Study design:Cross-sectional |
| Associations among physical activity, screen time, and sleep in low socioeconomic status urban girls | Greever 2017 | Study design:Cross-sectional |
| Association of Screen Time with Anxiety and Depressive Symptoms in College Students During COVID-19 Outbreak in Shanghai: Mediation Role of Sleep Quality | Liu 2023 | Study design:Cross-sectional |
| The Association of Soft Drink Consumption and the 24-Hour Movement Guidelines with Suicidality among Adolescents of the United States | Liu 2022 | Study design:Cross-sectional |
| Television Viewing in Infancy and Child Cognition at 3 Years of Age in a US Cohort | Schmidt 2015 | Data not meeting inclusion criteria |
| Mobile usage and sleep patterns among medical students | Yogesh 2014 | Study design:Cross-sectional |
| Mobile phone dependency and sleep quality in college students during COVID-19 outbreak: the mediating role of bedtime procrastination and fear of missing out | Huang 2023 | Study design:Cross-sectional |
| Association of Child and Adolescent Mental Health With Adolescent Health Behaviors in the UK Millennium Cohort | Hoare 2020 | Data not meeting inclusion criteria |
| Association between the pattern of mobile phone use and sleep quality in Northeast China college students | Meng 2021 | Study design:Cross-sectional |
| Associations of Parenthood with Physical Activity, Sedentary Behavior, and Sleep | Carson 2018 | Study design:Cross-sectional |
| Sleep Habits and Electronic Media Usage in Japanese Children: A Prospective Comparative Analysis of Preschoolers | Horiuchi 2020 | Study design:Cross-sectional |
| A study on the effect of mobile phone use on sleep | Sinha 2022 | Study design:Cross-sectional |
| Presence of Small Screens in Bedrooms Is Associated With Shorter Sleep Duration and Later Bedtimes in Children With Obesity | Duggan 2018 | Study design:Cross-sectional |
| Social media use in female adolescents: Associations with anxiety, loneliness, and sleep disturbances | Azhari 2022 | Study design:Cross-sectional |
| Extracurricular Activities, Screen Media Activity, and Sleep May Be Modifiable Factors Related to Children's Cognitive Functioning: Evidence From the ABCD Study | Kirlic 2021 | Data not meeting inclusion criteria |
| Morningness-eveningness correlates with sleep time, quality, and hygiene in secondary school students: a multilevel analysis | Vollmer 2016 | Study design:Cross-sectional |
| Dietary and Activity Factors Influence Poor Sleep and the Sleep-Obesity Nexus among Children | Morrissey 2019 | Study design:Cross-sectional |
| Exploring the impact of COVID-19 on the movement behaviors of children and youth: A scoping review of evidence after the first year | Paterson 2021 | Study design:Review |
| Socioeconomic position as a predictor of youth's movement trajectory profiles between ages 10 and 14 years | Wilhite 2023 | Data not meeting inclusion criteria |
| The role of sleep in the relation between young children's mobile media use and effortful control | Nathanson 2017 | Study design:Cross-sectional |
| The combined impact of diet, physical activity, sleep and screen time on academic achievement: a prospective study of elementary school students in Nova Scotia, Canada | Faught 2017 | Data not meeting inclusion criteria |
| Ecological Momentary Assessment of Physical Activity and Wellness Behaviors in College Students Throughout a School Year: Longitudinal Naturalistic Study | Bai 2022 | Data not meeting inclusion criteria |
| The home environment and childhood obesity in low-income households: indirect effects via sleep duration and screen time | Appelhan 2014 | Study design:Cross-sectional |
| Age, income and sleep duration were associated with outcomes in children participating in weight management | Kjetsa 2022 | Study design:RCT |
| Evening and night exposure to screens of media devices and its association with subjectively perceived sleep: Should "light hygiene" be given more attention? | Smotek 2020 | Study design:Cross-sectional |
| Paths towards a healthier BMI among short and adequate sleepers: A pathway network analysis considering movement behaviors in low-income preschoolers | Souza 2023 | Study design:Cross-sectional |
| Media use and child sleep: the impact of content, timing, and environment | Garrison 2011 | Study design:RCT |
| Examining the day-to-day bidirectional associations between physical activity, sedentary behavior, screen time, and sleep health during school days in adolescents | Kim 2020 | Study design:Cross-sectional |
| Long-term effect of mobile phone use on sleep quality: Results from the cohort study of mobile phone use and health (COSMOS) | Tettamanti 2020 | Exposure(did not meet inclusion criteria) |
| Sleep patterns in Spanish adolescents: associations with TV watching and leisure-time physical activity | Ortega 2010 | Study design:Cross-sectional |
| Quantity, Content, and Context Matter: Associations Among Social Technology Use and Sleep Habits in Early Adolescents | Charmaraman 2020 | Study design:Cross-sectional |
| Compositional associations of time spent in sleep, screen time, and physical activity with polysubstance use in adolescents | Zahran 2023 | Study design:Cross-sectional |
| A Longitudinal Analysis Examining the Associations of Tummy Time With Active Playtime, Screen Time, and Sleep Time | Buchanan 2021 | Data not meeting inclusion criteria |
| The use of entertainment and communication technologies before sleep could affect sleep and weight status: a population-based study among children | Dube 2017 | Study design:Cross-sectional |
| Sleeping, TV, Cognitively Stimulating Activities, Physical Activity, and Attention-Deficit Hyperactivity Disorder Symptom Incidence in Children: A Prospective Study | Gabriela 2017 | Data not meeting inclusion criteria |
| Parental Expectations Are Associated with Children's Sleep Duration and Sleep Hygiene Habits | Jarrin 2020 | Data not meeting inclusion criteria |
| Physical Activity, Screen Time, Sedentary and Sleeping Habits of Polish Preschoolers during the COVID-19 Pandemic and WHO's Recommendations: An Observational Cohort Study | Brzęk 2021 | Data not meeting inclusion criteria |
| Associations among Screen Time and Unhealthy Behaviors, Academic Performance, and Well-Being in Chinese Adolescents | Yan 2017 | Study design:Cross-sectional |
| Influence of physical activity, screen time and sleep on inmates' body weight during incarceration in Canadian federal penitentiaries: a retrospective cohort study | Johnson 2019 | Outcome (did not meet inclusion criteria) |
| Associations between screen time and sleep duration are primarily driven by portable electronic devices: evidence from a population-based study of U.S. children ages 0-17 | Jean 2018 | Study design:Cross-sectional |
| [Multi-center study on the effects of television viewing on sleep quality among children under 4 years of age in China] | Dong 2015 | Study design:Cross-sectional |
| Longitudinal associations between cyber victimization and problematic mobile phone use in adolescents: Disentangling between-person effects from within-person effects | Chen 2023 | Data not meeting inclusion criteria |
| Do young children consistently meet 24-h sleep and activity guidelines? A longitudinal analysis using actigraphy | Meredith-Jones 2019 | Data not meeting inclusion criteria |
| The effects of prolonged single night session of videogaming on sleep and declarative memory | Hartmann 2019 | Study design:Cross-sectional |
| Effect of maternal sleep, physical activity and screen time during pregnancy on the risk of childhood respiratory allergies: a sex-specific study | Chen 2020 | Outcome(did not meet inclusion criteria) |
| Impact of COVID-19 on the sleep-wake patterns of preschool children | Wong 2023 | Data not meeting inclusion criteria |
| Association between screen time and sleep habits in 11-to-12-year-old French middle school students | Messaadi 2020 | Study design:Cross-sectional |
| Association of screen time and sleep duration among Spanish 1-14 years old children | Cartanya-Hueso 2020 | Study design:Cross-sectional |
| Sleep and Psychological Functioning of Children and Adolescents - a Narrative Review | Brand 2019 | Study design:Review |
| Outdoor time, screen time and sleep reported across early childhood: concurrent trajectories and maternal predictors | Downing 2022 | Study design:RCT |
| Isotemporal Substitution Analysis for Sedentary Behavior and Body Mass Index | Huang 2016 | Outcome(did not meet inclusion criteria) |
| Association of mobile phone overuse with sleep disorder and unhealthy eating behaviors in college students of a medical university in Guangzhou] | Fang 2019 | Study design:Cross-sectional |
| Screen time and early adolescent mental health, academic, and social outcomes in 9- and 10- year old children: Utilizing the Adolescent Brain Cognitive Development ℠ (ABCD) Study | Paulich 2021 | Data not meeting inclusion criteria |
| Meeting 24-h movement guidelines and associations with health related quality of life of Australian adolescents | Khan 2020 | Data not meeting inclusion criteria |
| he Longitudinal Relationship Between Screen Time, Sleep and a Diagnosis of Attention-Deficit/Hyperactivity Disorder in Childhood | Levelink 2021 | Data not meeting inclusion criteria |
| Children's behavioral problems, screen time, and sleep problems' association with negative and positive parenting strategies during the COVID-19 outbreak in Brazil | Oliveira2022 | Study design:Cross-sectional |
| TV exposure associated with sleep disturbances in 5- to 6-year-old children | Paavonen 2006 | Study design:Cross-sectional |
| Day-to-day and longer-term longitudinal associations between physical activity, sedentary behavior, and sleep in children | Okely 2021 | Data not meeting inclusion criteria |
| Nighttime media use in adolescents with ADHD: links to sleep problems and internalizing symptoms | Becker 2018 | Study design:Cross-sectional |
| Beyond Access and Exposure: Implications of Sneaky Media Use for Preschoolers' Sleep Behavior | Moorman 2018 | Study design:Cross-sectional |
| Predictors of Changes in Sleep Duration in Dutch Primary Schoolchildren: the ChecKid Study | Komrij 2020 | Data not meeting inclusion criteria |
| Preschoolers' engagement with screen content and associations with sleep and cognitive development | Axelsson 2022 | Study design:Cross-sectional |
| The association between evening social media use and delayed sleep may be causal: Suggestive evidence from 120 million Reddit timestamps | Meyerson2023 | Study design:Cross-sectional |
| Sociodemographic differences in 24-hour time-use behaviours in New Zealand children | Hedayatrad 2022 | Data not meeting inclusion criteria |
| Associations between the Neighborhood Social Environment and Obesity Among Adolescents: Do Physical Activity, Screen Time, and Sleep Play a Role? | Saelee 2020 | Data not meeting inclusion criteria |
| Bedtime Electronic Media Use and Sleep in Children with Autism Spectrum Disorder | Mazurek 2016 | Study design:Cross-sectional |
| Physical activity, sedentary behaviour and sleep in Canadian children: parent-report versus direct measures and relative associations with health risk | Colley 2012 | Data not meeting inclusion criteria |
| Mobile phone use and stress, sleep disturbances, and symptoms of depression among young adults--a prospective cohort study | Thomee 2011 | Outcome:mental health |
| Internet overuse and excessive daytime sleepiness in adolescents | Choi 2009 | Study design:Cross-sectional |
| School start time changes in the COMPASS study: associations with youth sleep duration, physical activity, and screen time | Patte 2018 | Exposure:School start time |
| The Impact of Television, Electronic Games, and Social Technology Use on Sleep and Health in Adolescents with an Evening Circadian Preference | Gumport 2021 | Study design:Cross-sectional |
| Youth Screen Time and Behavioral Health Problems: The Role of Sleep Duration and Disturbances | Parent 2016 | Study design:Cross-sectional |
| Internet use and its impact on internalizing disorder symptoms and sleep in adolescents with an evening circadian preference | Lauren 2021 | Study design:Cross-sectional |
| Diet, Physical Activity, and Screen Time to Sleep Better: Multiple Mediation Analysis of Lifestyle Factors in School-Aged Children with and without Attention Deficit Hyperactivity Disorder | Hong 2020 | Study design:Cross-sectional |
| Impact of Media Use on Adolescent Sleep Efficiency | Aaron 2016 | Study design:Cross-sectional |
| Physical Activity, Screen Time, and Sleep in Children With ADHD | Tandon 2019 | Data not meeting inclusion criteria |
| Television-viewing habits and sleep disturbance in school children | Owens 1999 | Study design:Cross-sectional |
| Featured Article: Technology Use and Sleep in Adolescents With and Without Attention-Deficit/Hyperactivity Disorder | Bourchtein 2019 | No data |
| The moderating roles of bedtime activities and anxiety/depression in the relationship between attention-deficit/hyperactivity disorder symptoms and sleep problems in children | Tong 2018 | Study design:Cross-sectional |
| Sleep Patterns and Quality Are Associated with Severity of Obesity and Weight-Related Behaviors in Adolescents with Overweight and Obesity | Hayes 2017 | Study design:RCT |
| The impact of lockdown on sleep patterns of children and adolescents with ADHD | Bruni 2017 | Study design:Cross-sectional |
| Correlates of short sleep duration among adolescent | Widome 2019 | Data not meeting inclusion criteria |
| Correlates of nocturnal sleep duration, nocturnal sleep variability, and nocturnal sleep problems in toddlers: results from the GET UP! Study | Zhang 2018 | Study design:RCT |
| The association between use of electronic media in bed before going to sleep and insomnia symptoms, daytime sleepiness, morningness, and chronotype | Fossum 2013 | Study design:Cross-sectional |
| Trends in sleeping difficulties among European adolescents: Are these associated with physical inactivity and excessive screen time? | Ghekiere 2018 | Study design:Cross-sectional |
| Impact of singular excessive computer game and television exposure on sleep patterns and memory performance of school-aged children | Markus 2007 | Study design:RCT |
| Does Sleep Mediate the Association between School Pressure, Physical Activity, Screen Time, and Psychological Symptoms in Early Adolescents? A 12-Country Study | Vandendriessche 2019 | Study design:Cross-sectional |
| Environmental and behavioural factors associated with school children's sleep in Aotearoa/New Zealand | Muller 2016 | Exposure(did not meet inclusion criteria) |
| Social media use predicts later sleep timing and greater sleep variability: An ecological momentary assessment study of youth at high and low familial risk for depression | Hamilton 2020 | Study design:Cross-sectiona |
| The association between excessive screen-time behaviors and insufficient sleep among adolescents: Findings from the 2017 youth risk behavior surveillance system | Baiden 2019 | Study design:Cross-sectiona |
| Adolescent Sleep Barriers: Profiles within a Diverse Sample of Urban Youth | Hoyt 2018 | Study design:Cross-sectiona |
| The impact of media use on sleep patterns and sleep disorders among school-aged children in China | Li 2007 | Study design:Cross-sectiona |
| Fear of missing out and sleep: Cognitive behavioural factors in adolescents' nighttime social media use | Scott2018 | Study design:Cross-sectiona |
| Associations between screen time and short sleep duration among adolescents varies by media type: evidence from a cohort study | Hisler 2019 | Data not meeting inclusion criteria |
| Reducing the use of screen electronic devices in the evening is associated with improved sleep and daytime vigilance in adolescents | Perrault 2019 | Study design:Cross-sectiona |
| Factors associated with sleep duration among pupils | Kohyama 2020 | Study design:Cross-sectiona |
| Adolescent use of social media and associations with sleep patterns across 18 European and North American countries | Nissim 2023 | Study design:Cross-sectiona |
| Protective and risk factors associated with adolescent sleep: findings from Australia, Canada, and The Netherlands | Bartel 2016 | Study design:Cross-section |
| Sleep problems: predictor or outcome of media use among emerging adults at university? | Tavernier 2014 | Data not meeting inclusion criteria |
| Social media use and adolescent sleep patterns: cross-sectional findings from the UK millennium cohort study | Scott 2019 | Study design:Cross-section |
| [Longitudinal correlation between cell phone use and sleep quality in college students] | Zhang 2022 | Exposure(did not meet inclusion criteria) |
| Cohort effects associated with reduced sleep duration in adolescents | Yoo 2019 | Data not meeting inclusion criteria |
| Sleep duration and risk of physical aggression against peers in urban youth | Street 2016 | Study design:Cross-section |
| Technology Use and Sleep Quality in Preadolescence and Adolescence | Bruni 2015 | Study design:Cross-section |
| Screen use, sleep duration, daytime somnolence, and academic failure in school-aged adolescents | Chada 2023 | Study design:Cross-section |
| Digital Screen Time and Pediatric Sleep: Evidence from a Preregistered Cohort Study | Przybylski 2018 | Data not meeting inclusion criteria |
| Screen time exposure and sleep among children with developmental disabilities AIM: Children with developmental disabilities are at risk of excessive screen time and are more vulnerable to sleep problems. | Aishworiya 2018 | Study design:Cross-section |
| Adolescents' electronic media use at night, sleep disturbance, and depressive symptoms in the smartphone age | Lemola 2014 | Study design:Cross-section |
| Insufficient Sleep Duration Is Associated With Dietary Habits, Screen Time, and Obesity in Children | Tambalis 2018 | Study design:Cross-section |
| The use of social media modifies teenagers' sleep-related behavior | Royant-Parola 2018 | Study design:Cross-section |
| Adolescents’ screen time displaces multiple sleep pathways and elevates depressive symptoms over twelve months | Hökby 2025 | Data not meeting inclusion criteria |
| Sedentary Behaviors, Light-Intensity Physical Activity, and Healthy Aging. | Shi 2024 | Outcome(did not meet inclusion criteria) |
| Digital media use and sleep in late adolescence and young adulthood: A systematic review | Brautsch 2023 | Study design:Review |
| Longitudinal associations between digital media use and ADHD symptoms in children and adolescents: a systematic literature review | Thorel 2024 | Study design:Review |
| What we know about screen time and social media in early adolescence: a review of findings from the Adolescent Brain Cognitive Development Study | Nagata 2025 | Outcome(did not meet inclusion criteria) |
| Childhood Lifestyle Behaviors and Mental Health Symptoms in Adolescence | Haapala 2025 | Outcome(did not meet inclusion criteria) |
| Longitudinal associations of screen time, physical activity, and sleep duration with body mass index in U.S. youth | Zink 2024 | Data not meeting inclusion criteria |
| Role of Sleep and White Matter in the Link Between Screen Time and Depression in Childhood and Early Adolescence | Santos | Outcome(did not meet inclusion criteria) |
| Diet, Eating Habits, and Lifestyle Factors Associated with Adequate Sleep Duration in Children and Adolescents Living in 5 Mediterranean Countries: The DELICIOUS Project | Godos 2025 | ExposureOutcome(did not meet inclusion criteria) |
| Replacing screen time, with physical activity and sleep time: influence on cardiovascular indicators and inflammatory markers in Brazilian children | Reis,2024 | Study design:Cross-section |
| Association between changes in adherence to the 24-hour movement guidelines with depression and anxiety symptoms among Chinese adolescents: a prospective population-based study | Wu 2024 | Outcome(did not meet inclusion criteria) |
| Screen time and sleep duration in pediatric critical care: Secondary analysis of a pilot observational study | Beth 2024 | Study design:Cross-section |
| Lifestyle Clusters of Diet Quality, Sleep, and Screen Time and Associations with Weight Status in Children from Madrid City: ENPIMAD Study | Peral-Suarez 2024 | Study design:Cross-section |
| Physical Activity, Sedentary Behavior, Sleep and Screen Time of Healthy Under-Fives Attending Selected Immunization Clinics and Anganwadis of South Kerala, India | Sreelakshmi 2024 | Study design:Cross-section |
| Reevaluating Sleep Quality During COVID-19 and Associations With Quarantine, Mental Health, Stress, and Screen Time in Young Adults: Results From a Cross-Sectional Online Survey | Wiciak 2023 | Study design:Cross-section |
| Screen Time and Objectively Measured Sleep of U.S. College Students: A Brief Report | Sedaghat 2025 | Data not meeting inclusion criteria |
| Associations of physical activity, screen time, sleep duration with optimal eating habits among adolescents | Xiang 2025 | Study design:Cross-section |
| Associations Between Screen Time, Physical Activity, and Sleep Patterns in Children Aged 3–7 Years—A Multicentric Cohort Study in Urban Environment | Torres 2025 | Data not meeting inclusion criteria |
| Associations of media use behaviors by types and content with sleep problems in Chinese adolescents | Gao 2025 | Study design:Cross-section |
