# Supplementary material for: The association of screen time and the risk of sleep outcomes: a systematic review and meta-analysis
Source: Front Psychiatry. 2025 Dec 17;16:1640263. doi: 10.3389/fpsyt.2025.1640263 (PMC12754674; doi:10.3389/fpsyt.2025.1640263)
Supplement: Supplementary file 1 [file DataSheet1.zip › Supplementary Table 2.docx]

**Supplementary Table 2. Summary findings of screentime and sleep outcomes**

| Outcome | Studies, n | Effect size  (95%CI) | GRADE  assessment | *P-* interaction | I- square, % |
| --- | --- | --- | --- | --- | --- |
| Continuous Outcome | | | | | |
| Sleep duration | 11 | -0.05(-0.08 to -0.03) | Very Low | / | 61.3% |
| Insomnia | 2 | 0.41(0.18 to 0.63) | Low | / | 69.3% |
| Binary Outcome | | | | | |
| Sleep duration | 9 | 1.25(1.08 to 1.41) | Moderate | / | 96.5% |
| Subgroup (Continuous Outcome) * | | | | | |
| Country | | | | | |
| US | 4 | -0.03(-0.05 to -0.02) |  |  | 7.0% |
| Australia | 2 | -0.07(-0.11 to 0.04) |  |  | 0.0% |
| Other countries | 5 | -0.12(-0.29 to 0.05) |  |  | 78.0% |
| Overall | 11 |  | Low | 0.81 |  |
| Region | | | | | |
| Asia | 2 | -0.02(-0.21 to 0.16) |  |  | 0.0% |
| America | 4 | -0.03(-0.05 to 0,02) |  |  | 7.0% |
| Oceania | 3 | -0.05(-0.08 to -0.02) |  |  | 48.0% |
| Other region | 2 | -0.25(-0.74 to 0.24) |  |  | 89.8% |
| Overall | 11 |  | Low | 0.33 |  |
| Age | | | | | |
| <18 years old | 9 | -0.07(-0.11 to -0.03) |  |  | 68.7% |
| ≥18 years old | 2 | -0.03 (-0.05 to -0.01) |  |  | 0.0% |
| Overall | 11 |  | Low | 0.48 |  |
| Type of Population | | | | | |
| Infant | 2 | -0.04(-0.11 to 0.04) |  |  | 0.0% |
| Toddler | 2 | -0.08(-0.14 to -0.02) |  |  | 0.8% |
| Preschooler | 1 | -0.07(-0.12 to -0.02） |  |  |  |
| Child | 2 | -0.07(-0.12 to -0.02) |  |  | 0.0% |
| Adolescent | 2 | -0.25(-0.71 to 0.21) |  |  | 94.3% |
| Adult | 2 | -0.03(-0.05 to -0.01) |  |  | 0.0% |
| Overall | 11 |  | Low | 0.31 |  |
| Follow-up time | | | | | |
| < 2.5 years | 5 | -0.03(-0.04 to -0.02) |  |  | 0.0% |
| ≥2.5 years | 6 | -0.07(-0.13 to -0.01) |  |  | 76.3% |
| Overall | 20 |  | Low | 0.72 |  |
| Subgroup (Binary Outcome) | | | | | |
| Country | | | | | |
| US | 2 | 0.32(0.05 to 0.558) |  |  | 94.3% |
| China | 3 | 0.47(0.28 to 0.67) |  |  | 9.2% |
| Other countries | 4 | 0.07 (0.04to 0.10) |  |  | 9.9% |
| Overall | 9 |  | Low | 0.004 |  |
| Region | | | | | |
| Asia | 4 | 0.35(0.05 to 0.64) |  |  | 83.4% |
| America | 2 | 0.32(0.05 to 0.58) |  |  | 94.3% |
| Oceania | 1 | -0.03(-0.26 0.20) |  |  |  |
| Europe | 2 | 0.08(0.02 to 0.14) |  |  | 50.1% |
| Overall | 9 |  | Low | 0.099 |  |
| Age | | | | | |
| <18 years old | 6 | 0.23(0.08 to 0.31) |  |  | 98.2% |
| ≥18 years old | 3 | 0.23 (0.04 to 0.41) |  |  | 47.5% |
| Overall | 9 |  | Low | 0.81 |  |
| Type of Population | | | | | |
| Preschooler | 2 | 0.66(0.35 to 0.96） |  |  | 92.4% |
| Adolescent | 3 | 0.33(0.12 to 0.54) |  |  | 88.6% |
| Adult | 2 | 0.16(-0.01 to 0.33) |  |  | 228.5% |
| Other population | 2 | 0.06(0.03 to 0.09) |  |  | 0.0% |
| Overall | 9 |  | Low | 0.21 |  |
| Follow-up time | | | | | |
| < 2.7 years | 5 | 0.27(0.09 to 0.45) |  |  | 83.3% |
| ≥2.7 years | 4 | 0.17(-0.07 to 0.40) |  |  | 98.1% |
| Overall | 9 |  | Low | 0.46 |  |
| Short sleep definition | | | | | |
| Aligned with NSF/AASM guidelines | 4 | 0.37(0.14 to 0.56) |  |  | 67.6% |
| Not specified | 5 | 0.15(-0.04 to 0.33) |  |  | 98.5% |
| Overall | 9 |  | Low | 0.43 |  |
