# Supplementary material for: The association of screen time and the risk of sleep outcomes: a systematic review and meta-analysis
Source: Front Psychiatry. 2025 Dec 17;16:1640263. doi: 10.3389/fpsyt.2025.1640263 (PMC12754674; doi:10.3389/fpsyt.2025.1640263)
Supplement: Supplementary file 1 [file DataSheet1.zip › Supplementary Table 3.docx]

**Supplementary Table 3. Meta-regression variables**

| **Meta-regression variables** | **Outcomes-Continuous Outcome** | |  |
| --- | --- | --- | --- |
|  | **Sleep duration** | |  |
|  | **Effect（β）** | **95%CI** | **P-value** |
| **Country** | -0.004 | -0.045 to 0.036 | 0.813 |
| **Region** | -0.034 | -0.109 to 0.410 | 0.333 |
| **Age** | 0.034 | -0.070 to 0.137 | 0.478 |
| **Type of Population** | 0.009 | -0.010 to 0.028 | 0.313 |
| **Follow-up time** | -0.015 | -0.107 to 0.077 | 0.671 |
| **Meta-regression variables** | **Outcomes-Binary Outcome** | |  |
|  | **Sleep duration** | |  |
|  | **Effect（OR）** | **95%CI** | **P-value** |
| **Country** | -0.092 | -0.145 to -0.040 | 0.004 |
| **Region** | -0.118 | -0.265 to 0.029 | 0.099 |
| **Age** | 0.041 | -0.171 to 0.182 | 0.814 |
| **Type of Population** | 0.028 | -0.352 to 0.434 | 0.192 |
| **Follow-up time** | -0.115 | -0.462 to 0.232 | 0.458 |
| **Short sleep definition** | -0.168 | -0.643 to 0.305 | 0.427 |
